# Shifts of Gamma Phase across Primary Visual Cortical Sites Reflect Dynamic Stimulus-Modulated Information Transfer

**DOI:** 10.1371/journal.pbio.1002257

**Published:** 2015-09-22

**Authors:** Michel Besserve, Scott C. Lowe, Nikos K. Logothetis, Bernhard Schölkopf, Stefano Panzeri

**Affiliations:** 1 Max Planck Institute for Biological Cybernetics, Tübingen, Germany; 2 Max Planck Institute for Intelligent Systems, Tübingen, Germany; 3 Institute for Adaptive and Neural Computation, School of Informatics, University of Edinburgh, Edinburgh, United Kingdom; 4 Division of Imaging Science and Biomedical Engineering, University of Manchester, Manchester, United Kingdom; 5 Laboratory of Neural Computation, Center for Neuroscience and Cognitive Systems @UniTn, Istituto Italiano di Tecnologia, Rovereto, Italy; Yeshiva University Albert Einstein College of Medicine, UNITED STATES

## Abstract

Distributed neural processing likely entails the capability of networks to reconfigure dynamically the directionality and strength of their functional connections. Yet, the neural mechanisms that may allow such dynamic routing of the information flow are not yet fully understood. We investigated the role of gamma band (50–80 Hz) oscillations in transient modulations of communication among neural populations by using measures of direction-specific causal information transfer. We found that the local phase of gamma-band rhythmic activity exerted a stimulus-modulated and spatially-asymmetric directed effect on the firing rate of spatially separated populations within the primary visual cortex. The relationships between gamma phases at different sites (phase shifts) could be described as a stimulus-modulated gamma-band wave propagating along the spatial directions with the largest information transfer. We observed transient stimulus-related changes in the spatial configuration of phases (compatible with changes in direction of gamma wave propagation) accompanied by a relative increase of the amount of information flowing along the instantaneous direction of the gamma wave. These effects were specific to the gamma-band and suggest that the time-varying relationships between gamma phases at different locations mark, and possibly causally mediate, the dynamic reconfiguration of functional connections.

## Abstract 2

This study reveals the presence of gamma-frequency travelling waves, which appear to dynamically route the flow of information within the visual cortex.

## Author Summary

Complex and flexible behavior likely results from the ability of groups of neurons in the brain to reconfigure dynamically the information flow across different brain areas, depending on what the brain is engaged in (processing a stimulus or carrying out a task). Here, we investigate how oscillations of cortical activity in the gamma frequency range (50–80 Hz) may influence dynamically the direction and strength of information flow across different groups of neurons. By recording neural activity and measuring information flow between multiple locations in visual cortex during the presentation of Hollywood movies, we found that the arrangement of the phase of gamma oscillations at different locations indicated the presence of waves propagating along the cortical tissue. These waves were observed to propagate along the direction with the maximal flow of information transmitted between neural populations. The gamma waves changed direction during presentation of different movie scenes, and when this happened, the strength of information flow in the direction of the gamma wave propagation was transiently reinforced. These findings suggest that the propagation of gamma oscillations may reconfigure dynamically the directional flow of cortical information during sensory processing.

## Introduction

Visual cortical processing is highly distributed and likely depends crucially upon the cooperative interactions between different groups of neurons. Although there is an extensive knowledge about how individual neurons encode specific features of visual stimuli [1,2], how groups of neurons cooperate to give rise to a coherent perception of naturalistic scenes is still largely unknown [3,4]. One factor governing interactions among groups of neurons is their pattern of anatomical connections. This pattern in the primate primary visual cortex (V1) includes a local recurrent microcircuitry involving both inhibitory and excitatory neurons [5,6], as well as a larger network of horizontal connections spreading over several millimeters [1,7–11]. Such recurrent connectivity likely serves as an anatomical substrate to establish transient dynamic patterns of functional connectivity [12–15], allowing selective communication between the populations of neurons involved in visual function [16–19]. However, the physiological mechanisms that may transiently modulate the effective strength of any given connection are largely unknown.

One possibility is that transient interactions between neuronal groups depend upon the relative phase of the synchronization of gamma-band oscillations within each group [20–23]. Despite the growing support for a role of the relative temporal alignment of gamma oscillations in mediating communication, many questions about how they may operate remain unsolved [24]. In particular, while previous evidence linked gamma oscillations to symmetrical interactions between populations through synchronization [25], it is not known whether they can establish a directional communication within a brain area and modulate it transiently according to the needs of stimulus processing or the demands of the task. Finding a mechanism for dynamic routing of information is of major importance for understanding intracortical communication.

One reason why the above questions have not yet been fully clarified is that most previous studies considered symmetric measures of correlation (or synchronization) between neural populations [26], which cannot provide information about the direction of interaction. We thus introduce here nonlinear information theoretic tools to quantify directed communication and its modulation by the stimulus to assess the role of the phase of gamma oscillations in modulating dynamically the routing of information and to analyze how this routing relates to the spatiotemporal pattern of gamma phase [27,28]. We analyze spiking activity and Local Field Potentials (LFPs) simultaneously recorded from locations separated by up to a few millimeters in the V1 of macaques during the presentation of naturalistic color movies. These stimuli present spatially extended visual features varying over a wide range of ecologically relevant time scales and are therefore ideally suited to both dynamically coactivate groups of neurons processing different regions of the visual field and to investigate how interactions among active groups of neurons may be modulated by changes in stimuli.

We found that the local phase of gamma-band rhythmic activity exerts a dynamic, stimulus-modulated spatially asymmetric effect on the firing rate of spatially separated populations within V1 in a way that strongly suggests that directional information transfer is mediated by propagation of gamma oscillations. Further, differences in gamma phase across sites are transiently modulated by the visual stimulus (with propagation of waves from the sending site accentuated when the sending site is strongly stimulated by the visual stimulus in its receptive field [RF]), despite the absence of reliable locking of gamma phase to the stimulus at any individual site. Finally, transient changes of phase differences across sites (or spatial phase shifts) co-occurred with changes in the causal interactions exerted by gamma oscillations onto spiking activity at other sites. These findings suggest that the dynamical relationships between gamma phases at different locations mark, and possibly causally mediate, the dynamic reconfiguration of functional and effective network connections during information processing.

## Results

### Extracellular Multi-electrode Recordings in V1 during Movie Stimulation and in Absence of Visual Stimulation

**Fig 1:**
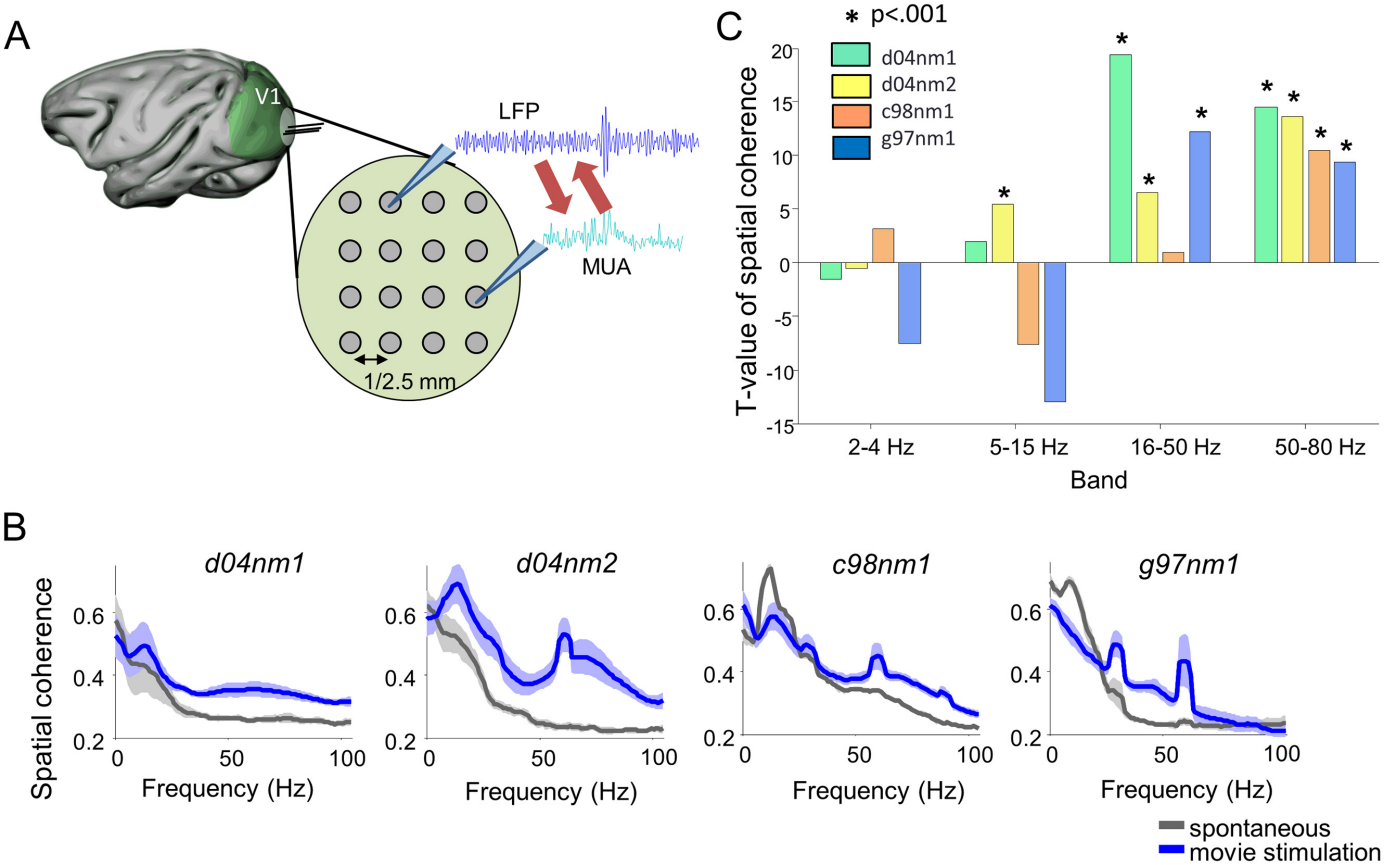
Extracellular multisite recordings in V1 during movie stimulation and spontaneous activity. (A) Scheme of the electrode configuration for the recording of LFPs and MUA in the V1 of anesthetized macaques, we study relationships between signals from distinct recording sites. (B) Spatial coherence of the LFPs averaged across experiments for each of the four recording sessions (d04nm1, d04nm2, c98nm1, g97nm1). Grey lines show spatial coherence computed during spontaneous activity (i.e., the screen used for visual stimulation was blank). Blue lines show spatial coherence during the response to visual stimulation with Hollywood movies. Shaded areas indicate the standard deviation (SD) across experiments. Coherence values were estimated using the multitaper approach applied on nonoverlapping 1 s duration blocks. (C) T-statistics of the comparison of LFP spatial coherence during movie stimulation with respect to spontaneous activity for different frequency bands and recording sessions. Stars indicate significantly positive values (*t* test, *p* < .001, Bonferroni corrected). Data available at http://dx.doi.org/10.6084/m9.figshare.1460872.

We recorded extracellular potentials in opercular V1 (foveal and parafoveal representations) of three anesthetized macaque monkeys with multiple electrodes positioned with a guide according to a 4x4 square grid with interelectrode spacing in the range 1–2.5 mm (Fig 1A). From the extracellular potentials recorded at each electrode, we extracted two aspects of mesoscopic network activity. First, we extracted Multiple Unit Activity (MUA) by filtering the extracellular signal in the (1,000–3,000 Hz) frequency range and computed the time-varying envelope of this oscillation. This signal is known to reflect the spike rate of neurons within 300 μm distance around the electrode tip [29]. The MUA was used here to measure the massed firing rate (and thus the strength of local network activity) at a given time and location. Second, we extracted LFPs by low-pass filtering the despiked extracellular potentials (see Materials and Methods). Here, LFPs, which are known to provide a robust measure of network oscillations [30,31], were used to measure the instantaneous phase and amplitude of network oscillations around the electrode location, using the Hilbert transform of the LFP band-passed signal (S1 Methods).

Activity was recorded both during binocular visual stimulation with 4–6.5 min long color Hollywood movie clips with 30 Hz frame rates (with the same movie clip being presented over 30–120 repeated trials in the same session) and during several 5 min long stretches without visual stimulation (spontaneous activity). RFs were identified for each site using reverse correlation of the gamma power (Materials and Methods, S1A and S1B Fig). RF distances for all electrode pairs were in the 0–4° range, and the majority of RF pairs had an overlap, with 46% of the data having a relative area overlap of 0.4 or less (S1C and S1D Fig). To study neural tuning to visual features, we extracted visual features from the RFs (primarily Orientation Activation—OA—and local Time Contrast—TC—see S1 Methods) with computer algorithms. The correlation over time of visual features (S1F Fig) showed that, though most RFs had a positive correlation, due to their partial overlap, the visual features in different RFs were partly independent (mean correlation 0.73 +/− 0.21 for TC, 0.4 +/− 0.30 for OA), thereby allowing some evaluation of how differences in visually-driven RF activation may modulate mesoscopic neural signals. The power spectrum of the changes of RF visual features over time (S1E Fig) showed that—in agreement with previous analyses of natural movies [32]—features varied slowly, with the most power in the low frequency range. Importantly, the properties of the presented movie imply that the gamma-band (50–80 Hz) oscillations cannot simply reflect the entrainment from stimulus dynamics and must originate instead from neural interactions.

Previous work showed that the LFP gamma-band was the one whose power carried more information about the movie stimulus ([33], see also S2B Fig), and whose power was proportionally more enhanced during movie stimulation with respect to spontaneous activity (S3 Fig, see also [34–36]). To assess in which LFP frequency band visual stimulation elicited spatially organized oscillatory activity, we computed the spatial coherence of the multisite recordings [37]. This measure has been previously used to detect interesting spatiotemporal activity such as travelling waves [37,38]. We found (Fig 1B and 1C) that visual stimulation elicited a consistent increase of spatial coherence in all sessions (*t* test; *p* < 0.001) only in the (50–80 Hz) gamma-band, suggesting that sensory information elicited spatially organized oscillations in this band (note the 60 Hz sharp coherence peaks correspond to harmonics of the 30 Hz frame rate of the movie and of the 60 Hz refresh rate of the monitor). Our aim was to investigate whether and how this stimulus-enhanced spatiotemporal coherent neural activity in the gamma-band mediates interactions and communication among neural populations at different locations.

### Information Theoretic Measures of the Impact of Gamma Phase on Neural Activity at Other Locations

Relationships between gamma phases of different neural populations have been reported to influence mutual, symmetric, relationships between the firing of different neuronal populations [26,39]. To extend this understanding to the case of directed—rather than mutual—information exchanges, here we investigated whether the phase of gamma oscillations of a neural population has a directed effect onto the firing rate of other receiving populations.

**Fig 2:**
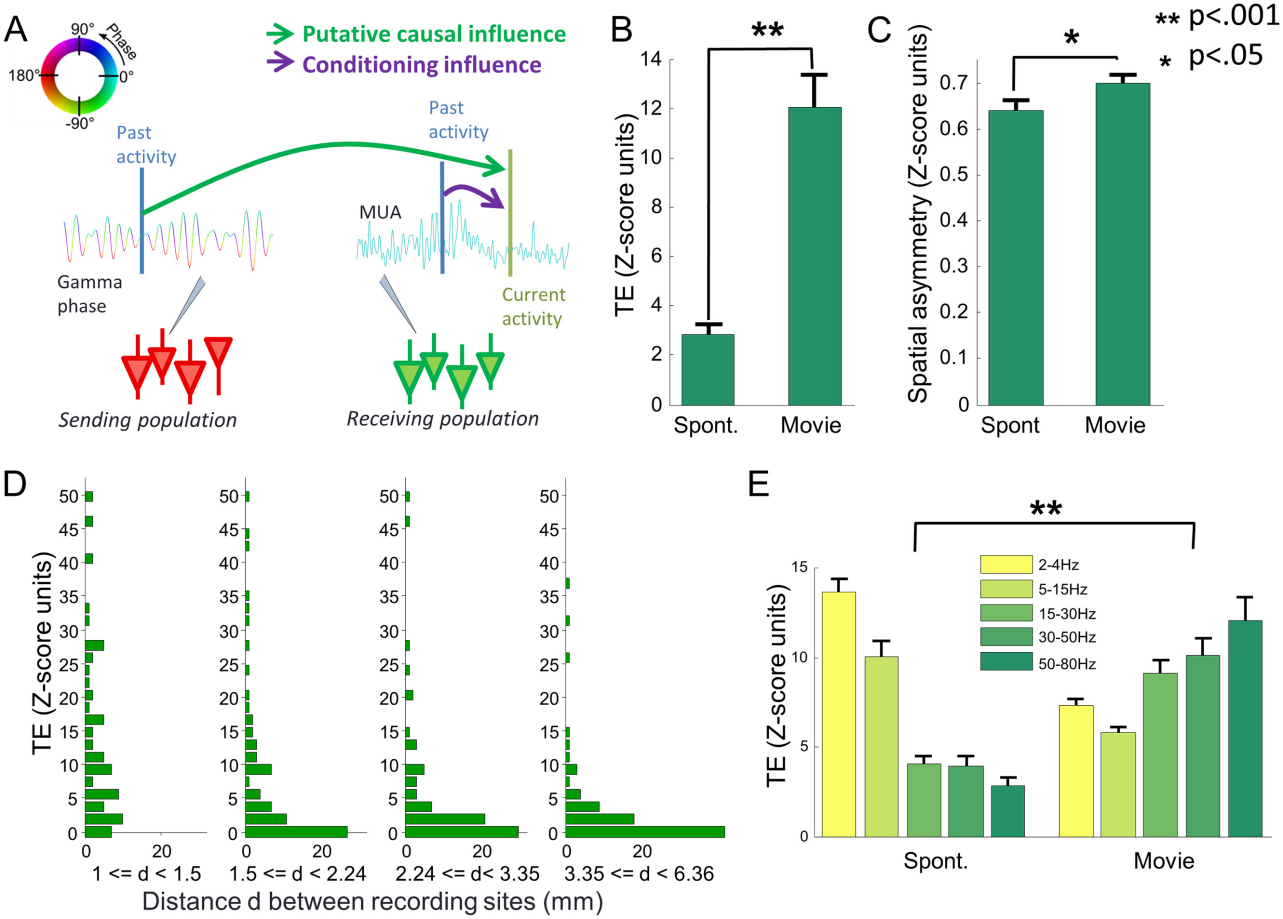
Causal impact of gamma phase on neural activity at other locations. (A) Principle of the calculation of TE quantifying the influence of phase at a “sending” recording site on the spiking activity at another “receiving” site. TE quantifies the information carried by the past values of the gamma phase at the sending site (the putative causal variable) about the current spiking activity at the receiving site (green arrow), while conditioning on the value of the past spiking activity at the receiving site (purple arrow). (B) TE values (Z-score units) averaged across all pairs of electrodes and sessions, during spontaneous activity and movie stimulation. Error bars indicate standard error (SE). Stars indicate significant increases during movie stimulation with respect to spontaneous activity (*t* test). (C) Spatial asymmetry ratio of TE values averaged across all couples of electrodes and sessions, during spontaneous activity and movie stimulation. Error bars indicate SE. (D) Histogram of TE values across all pairs of electrodes in all sessions, cropped to the 95th percentile. The electrode pairs were divided into four equipopulated ranges of interelectrode distances. (E) TE values (averaged across all pairs of electrodes in all sessions) quantifying the causal impact of phases of several frequency bands (whose frequency range is indicated in figure legend) during spontaneous activity and movie stimulation. Error bars indicate SE. Data available at http://dx.doi.org/10.6084/m9.figshare.1460872.

To address this question quantitatively, we used a direction-specific information theoretic analysis of the impact of phase on spiking activity at other sites during visual stimulation with natural movies. We measured whether the gamma phase of the sending population influences the spiking activity of the receiving population above and beyond what can be predicted by the past firing rate dynamics of the receiving population itself. That is, we computed the mutual information between the past gamma phase at a “sending” electrode and the spiking activity at a “receiving” electrode, conditioned upon the past spiking activity of the receiving population (see illustration in Fig 2A). This quantity is called the Transfer Entropy (TE) from the gamma phase at the sending electrode to the spiking activity at the receiving electrode [28]. Significantly positive values of TE mean that the gamma phase of the sending population exerts a causal effect (in the Wiener-Granger sense) on variations in firing rate in the receiving location. Note that TE values are reported after subtracting out spurious amounts of causation due to effects including common visual stimulation that do not reflect genuine communication between sites and are Z-scored in SD units of this spurious magnitude of causation (see S1 Methods and [40]). This TE analysis was performed for all available pairs of electrodes in each session, thereby running through all possible combinations of putative “sending” and “receiving” populations. Importantly, in this section and unless otherwise stated, we considered the overall information carried during the whole time of movie presentation. The analysis of how this is dynamically modulated during the presentation of the movie will be left for later sections. Mean values of TE across the entire dataset are reported in Fig 2B and show a high amount of TE (with Z-scored values of the order of 8–10 significant at *p* < 10^−7^; *t* test) from the gamma phase at the sending electrode to the spiking activity at the receiving electrode. TE values were significantly larger than zero (using a *t* test and a False Discovery Rate control with *q* = .05) for 84% of electrode pairs. Moreover, there was a more than 2-fold highly significant (*p* < .001; *t* test across pooled electrode pairs of all sessions) increase in TE magnitude during movie stimulation with respect to spontaneous activity (Fig 2B), suggesting these directed causal influences of phase relate to the processing of visual information.

We then investigated whether gamma phase of the sending population has an effect on the firing of the receiving population that goes above and beyond the one exerted by the firing rate of the sending population. To quantify this, we computed the Lagged Conditional Information (LCI), which is the mutual information between the past gamma phase at the sending electrode and the spiking activity at the receiving electrode, conditioned upon the past spiking activity of the sending population. The results (S4 Fig) showed a highly significant (with Z-scored values of the order of 8–10 significant at *p* < 10^−7^; *t* test) LCI, showing that the relationship between gamma phase of the sending population and the spiking activity of the receiving population cannot be accounted for by the relationship between spiking activity and gamma phase at the sending location.

To further corroborate this conclusion, we computed how correlated across all pairs of electrodes were the values of TE from the gamma phase of the sending electrode to the spiking activity of the receiving electrode with the TE from the spiking activity of the sending electrode to the spiking activity of the receiving electrode. We found that there was a significant (*p* < .05) but very small and negative correlation (Pearson ρ = −.12), again supporting the conclusion that gamma phase of a population exerts an effect on the firing rate of another receiving population that is largely different from that exerted by its firing rate.

Given that TE is a directed and potentially asymmetric measure that can detect a leading direction of communication, we quantified the degree of spatial asymmetry in our information measures. We define the spatial asymmetry index as the ratio between the absolute value of the difference in TE in both directions and the maximal information in one of the two directions. This index takes values from 0 to 1, with near-zero values indicating perfect symmetry and near-one values indicating prevalence of one-directional communication (See S1 Methods). In our dataset, we found that (Fig 2C) both during spontaneous activity and movie stimulation, the average asymmetry index was large—in the range 0.6–0.7, suggesting a prevalence of directed asymmetric effects of gamma phase on spiking activity of other sites over symmetric communications such as mutual interactions. Moreover, this asymmetry increased significantly during movie stimulation (*p* < .05; *t* test across pooled electrode pairs across sessions).

We next investigated how these directed interactions depend on the distance between recording sites. Fig 2D reports the histogram of TE values of the pairs of electrodes across all sessions. The data were partitioned into four equipopulated ranges of interelectrode distances. While a larger number of high TE values could be observed at short distances (<2.24 mm), several pairs with large interelectrode distances also exhibited large interactions. In addition, electrode pairs exhibiting very low values could be found at all distances, supporting that the TE is not a simple function of distance and arguing against that they could be ascribed to external artifacts or volume conduction. We further checked how asymmetry of causal interactions is influenced by distance by selecting pairs with both a sufficiently strong causal interaction (we eliminated in each session the 20% of electrodes pairs with the lowest TE values) and a direction of dominant causal interaction (one direction of causation 10 times bigger than the opposite direction). We call the so-defined pairs “strongly asymmetric pairs” (or in short asymmetric pairs). The distribution with respect to interelectrode distance of such strongly asymmetric pairs (S1 Table) shows that they are proportionally more frequent at larger distances, whereas “symmetric pairs” (defined as pairs whose relative difference between TE values of both directions is less than 20%, also eliminating the 20% of electrodes pairs with the lowest TE values) are proportionally more frequent at shorter distances.

Finally, we studied how frequency-specific is the causal influence of the phase of the sending population onto the spiking activity of the receiving population. To do so, we band-passed the LFP into four other lower-frequency bands (2–4 Hz, 5–15 Hz, 15–30 Hz, and 30–50 Hz), we computed the instantaneous phase and repeated the same TE analysis for the phase of each band. Comparisons of results across bands (Fig 2E) show that, although lower frequency bands had a larger causal effect during spontaneous activity, during visual stimulation with Hollywood movies the highest information values were obtained for the gamma-band. This suggests that gamma-band has an important role in transmitting and routing across sites the information needed for stimulus processing.

Overall, our information theoretic measures of interactions suggest that during stimulation with naturalistic movies, gamma phase of a primary visual cortical population exerts a genuine directed effect on the level of firing rate at other receiving locations within V1, and that this effect goes beyond what may be due to the level of firing rate at the receiving or the sending location.

### Phase Shifts across Electrodes Correlate with the Leading Direction of Causation and Can be Described As Travelling Waves

Previous studies suggested that symmetric mutual interactions among neural populations depend on the phase relationships between the rhythmic activity of the interacting neural populations rather than on the phase of one population only [26]. In the light of these observations, we asked the following questions: does the directed causal effect of the phase of gamma oscillations of a neural population onto the activity of other receiving populations depend on the phase relationships of gamma oscillations at different sites? If so, what are the specific phase relationships that correspond to larger causal effect of gamma phase on spiking activity at other locations?

**Fig 3:**
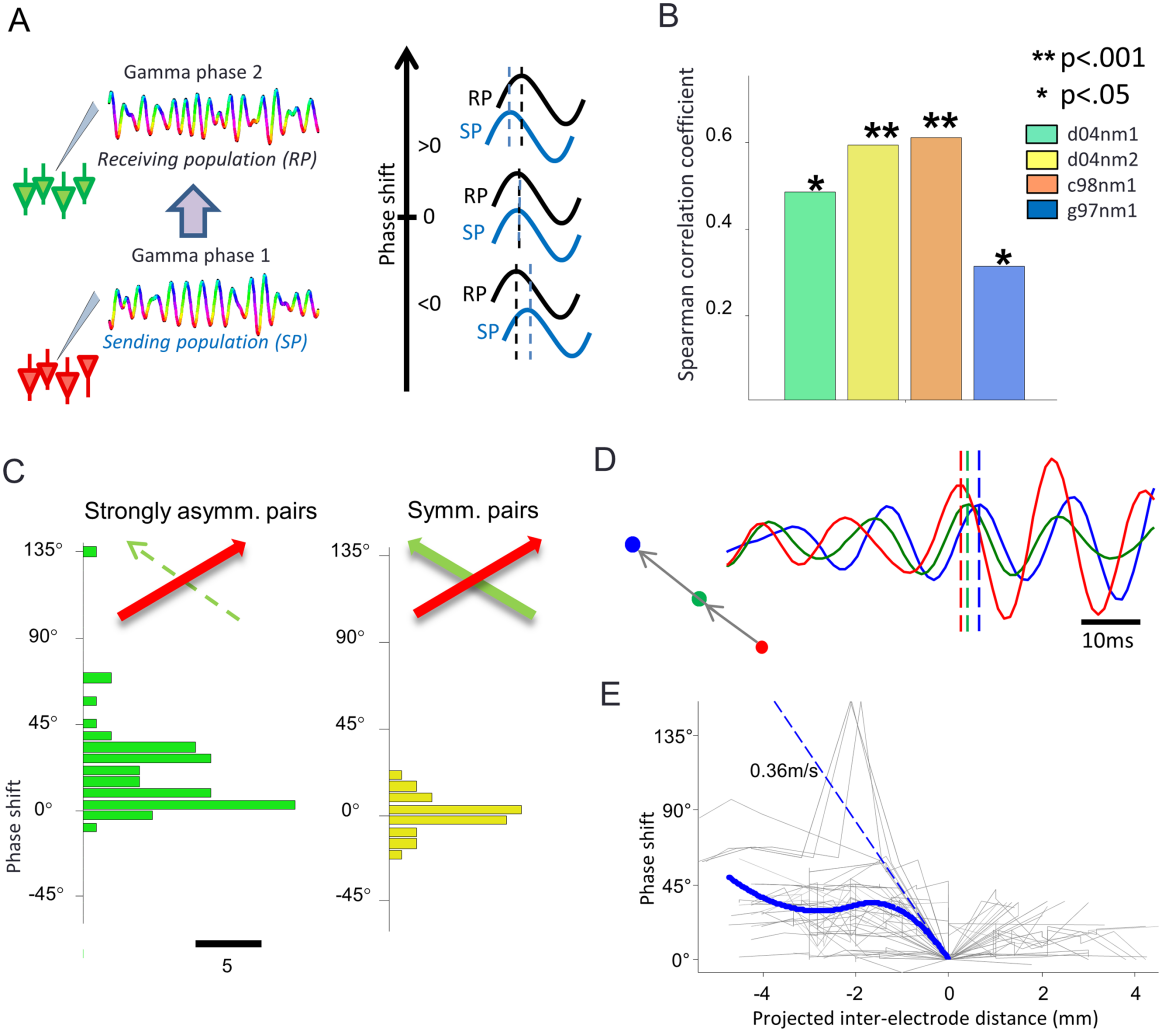
Spatial phase shifts. (A) Illustration of the meaning of the sign of the phase shift under our conventions. The ordering of the electrodes used to compute phase differences is illustrated on the left. At each time point, we compute the phase of the gamma-band-passed LFP in the receiving electrode, and we subtract it from the value of the phase of the gamma-band-passed LFP at the sending electrode. The cartoon on the right illustrates that positive (respectively negative) phase shifts indicate that the gamma oscillation at the sending side leads (respectively lags behind) the gamma oscillation at the receiving site. (B) Spearman’s correlation coefficient between the gamma phase shift and TE transferred between gamma phase at the sending site and MUA at the receiving site, computed separately for each recording session. Stars denote significant values. (C) Mean phase shift histogram for two groups of electrode pairs: on the left for strongly asymmetric pairs (shift is measured in the leading direction of causation). On the right, the histogram for symmetric pairs is shown. (D) Example of simultaneously-recorded LFP traces in the gamma-band for three aligned recording sites. Each site is color-coded. As schematized by the arrows in the left hand side, both blue and green recording sites were receiving sites, forming a strongly asymmetric pair with the sending recording site in red. During this time period, the time courses of each electrode (each plotted with the color of the corresponding electrode) exhibit ordered phase shift whose sign is consistent with wave propagation from the sending site to the receiving ones (see location of maxima corresponding to the zero phase of each signal, indicated by colored vertical bars). (E) Phase shift against projected interelectrode distance along the direction of causation. Gray lines indicate plots for individual directions of dominant propagation in all sessions. Approximation by cubic spline regression of the pooled data is shown as a solid blue line. The dashed blue line is the tangent of this regression function at the origin. The slope of the tangent is indicated. Data available at http://dx.doi.org/10.6084/m9.figshare.1460872.

To address these questions, we investigated the relationship between the spatiotemporal distribution of gamma phases and directed information transfer. We defined instantaneous phase shifts between two electrodes as the difference at each time point between the instantaneous gamma phases computed from the band-pass-filtered LFP at each electrode, and we quantified the circular mean across time of these phase shifts (see S1 Methods). To relate phase shifts to our measure of information transfer, we used the convention of measuring them as the difference between the phase at the “sending” electrode and the phase at the “receiving” electrode (the electrode at which the effects on MUA activity are considered), exactly as defined above when quantifying TE across sites. With this definition, a positive phase shift means that the oscillation in the sending electrode precedes the oscillation in the receiving electrode (see Fig 3A for an illustration).

In this section, we first considered the phase shift averaged over the entire time of movie presentation in all trials. (The dynamical changes of phase shifts over the time of movie presentation and their relationship to dynamic changes of information transfer will be addressed in later sections). Most electrode pairs showed absolute values of movie-averaged phase shifts distributed between 0° and 80° (S5 Fig). Moreover, 100% of the electrode pairs had a significantly nonuniform distribution over time of phase shifts (*p* < 0.01; Rayleigh test), meaning that the phase relationships among all electrodes were not random.

To test the relationship between phase differences and causal effects of gamma phases onto receiving sites, we computed the Spearman correlation between the movie-averaged phase shift of an electrode pair and the amount of causal effect (TE) exerted by gamma phase of the sending population onto spiking activity at the receiving electrode. The results (Fig 3B) show—consistently in all sessions—a significant (*p* < 0.05 with Bonferroni correction) positive Spearman correlation of phase shifts with TE. In other words, positive (respectively negative) phase differences corresponded to higher (respectively lower) values of TE between gamma phase at the sending location and spike rates at the receiving location. This is further illustrated by computing the histograms (cumulated across sessions) of the movie-averaged phase shifts for the above defined strongly asymmetric pairs. The result (Fig 3C) shows mostly positive phase differences. We found that the above-defined symmetric pairs had mean phase shifts distributed around the value 0°, corresponding to a zero-lag synchrony (Fig 3C).

A simple, yet accurate way to summarize these results is that the sign of the phase shifts indicates the dominant direction of interaction. In other words, causation predominantly flows from the location with the earlier phase to the location with the later phase. This pattern of phase differences is thus consistent with a simple compact description of the causal interactions as a propagating gamma wave.

We finally checked whether the relationships between phase differences and the magnitude and direction of causal effect of phase of rhythmic activity were specific to the gamma-band. We performed the same correlation analysis on the phase of LFP bands of frequencies lower than the gamma range (the 2–4 Hz, 5–15 Hz, and 16–50 Hz bands). As shown in S6 Fig, the correlations between the phase shifts and the TE between lower frequency phases and spiking activity were weaker (and not significant in all sessions) than the one found for the gamma phase, suggesting that the relationships between phase shifts and the magnitude and direction of causal effects of the phase of rhythmic activity were specific to the gamma-band.

### Propagation Speed of Putative Travelling Waves and Similarity of Orientation Selectivity of Sites with Strong Causal Interactions

Given that phase shifts indicate the direction of causation in neural activity and that they can be described as travelling waves, it is tempting to speculate that these shifts are associated to a propagation of gamma oscillations along the horizontal connections of V1. In the following, we investigated the extent to which these phase shifts are compatible with known physiological and anatomical properties of lateral connectivity.

Propagation of waves across space and time can be investigated by analyzing the patterns of phase differences across electrodes [37,38,41]. As illustrated in an example from our data (Fig 3D), if direction of gamma causation and gamma wave propagation are aligned, recording sites positioned along a line of prevalent TE flow (i.e., recordings sites that have asymmetric TE with their neighbors all pointing to a leading direction of causation) should also have gamma-band time lags and phase shifts distributed according to their algebraic positions along the direction of propagation. We estimated from this pattern the putative speed of propagation. Since the speed of a propagating wave is inversely proportional to the spatial derivative of the phase along the direction of propagation, we estimated the spatial phase shifts values against the distance along the directions defined by strongly asymmetric pairs in each session (assuming these pairs are prone to be oriented along the direction of propagation). For each single strongly asymmetric pair, designated as the “reference causal pair”, we studied the propagation along the line passing through both electrodes of this pair. We thus computed the phase shift between the receiving site of the considered reference causal pair (for the leading direction of causation) and all the other recording sites lying along the propagation axis defined by the line crossing both sites of the considered reference causal pair (see S1 Methods and S7 Fig). The algebraic propagation distance corresponding to each measured phase shift values was computed by projecting the interelectrode distance over the axis defined by the reference causal pair. The origin of the *x*-axis indicates the position of the receiving site. When the receiving site was not achieving a minimum (zero) phase shift with respect to the other electrodes, but instead this minimum was achieved by the receiving site of another strongly asymmetric pair, this latter receiving site was chosen as the reference of the *x*-axis, such that the origin always indicates the final target of the wave propagation, and not an intermediate site lying on the propagation trajectory. The results for each reference causal pair are plotted on Fig 3E in gray. To estimate the spatial derivative of the phase, these data points were used to fit a spline regression model (Fig 3E, in blue), showing a steep negative slope close to the origin and a progressive flattening further away from the origin. The decrease in the slope might reflect that the waves quickly attenuate as they propagate along the cortical surface; alternatively, it can possibly arise from interferences between travelling waves propagating on overlapping parts of this surface. To minimize those effects in the speed estimation, we estimate the speed of propagation when it is closest to its target receiving population located at the origin in our representation. We thus estimated the propagation speed from the spatial derivative of the phase where it is larger: at null interelectrode distance, using spline interpolation (see S1 Methods), leading to an average propagation speed of 36 ± 4 cm/s (mean ± bootstrap estimated SD). This propagation speed is similar in magnitude to the signal propagation speed along axons of excitatory horizontal connections reported in the literature [42–45].

We also investigated whether causal interactions were more prominent among pairs with similar orientation preference. For this, we first estimated the RF of each recording site by reverse correlation using the responses to the movie. We then estimated, by extracting the orientation content of each movie frame and correlating it with neural activity, the orientation tuning curve of multiunit spiking activity at each recording site (see [46,47] and Materials and Methods). The Spearman correlation between orientation tuning similarity (measured by covariance between the curves) and TE, across all electrode pairs from all sessions was positive (ρ = 0.24, *p* < 0.01). Since horizontal connections are more likely among populations with similar orientation preferences [11,48,49], this finding is compatible with the hypothesis that gamma phase shifts may reflect causal interactions propagating along horizontal connections.

### Phase Shifts Can be Dynamically Modulated by the Stimulus

The above findings demonstrated that the direction and strength of the causation exerted by gamma phase on spiking activity at other receiving sites correlates, on average, over long periods of dynamic visual stimulation, with the difference in gamma phase at both sites. It has been suggested that patterns of phase relationships may act as a dynamical gain factor that weights the effect of the anatomical connection infrastructure and therefore allows to modulate rapidly the strength of interactions among populations [23]. Evidence in support of this theory has been reported at the level of mutual symmetric interactions [26]. To understand whether this dynamical modulation may apply also to the case of directed interactions documented here, we asked whether changes in gamma phase shifts are related to changes in the stimulus, and whether they are accompanied by a readjustment of the magnitude of causal interactions among sites. In this section, we begin by studying whether gamma phase shifts can be dynamically and reliably modulated by the stimulus.

**Fig 4:**
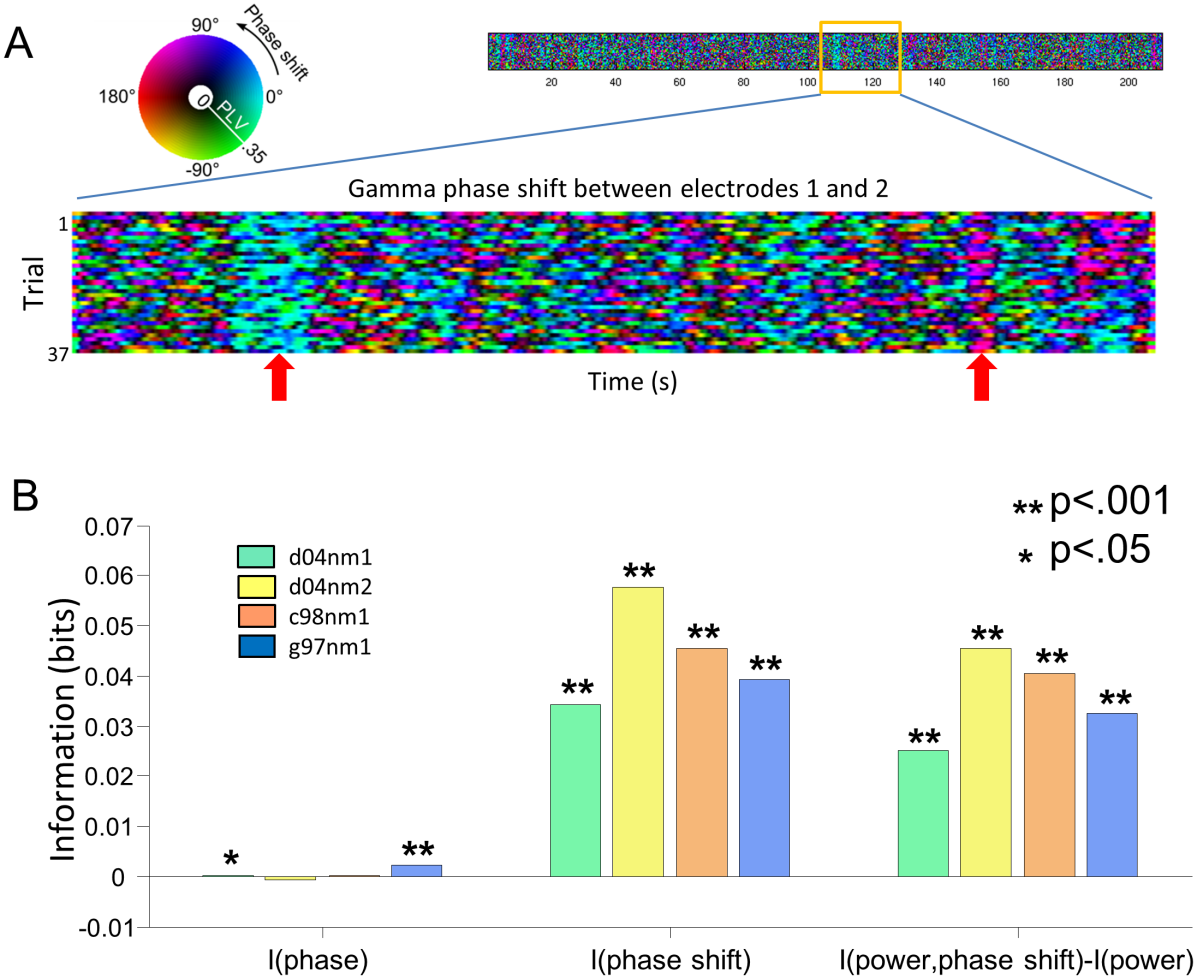
Shifts of gamma phase are dynamically modulated by the visual stimuli. (A) Example of the temporal dynamics of phase shifts for an example electrode pair (from session d04nm2), smoothed (with circular average) over 300 ms sliding windows. Hue encoding is the same as in Fig 3A. In addition, intensity encodes PLV (see text). The red arrows indicate periods of reliable modulation of the phase difference by the stimulus. (B) Sensory information carried by several features of the gamma oscillations averaged across pairs of electrodes for each session. Left and center bars indicate the sensory information carried by gamma phase and gamma phase shifts, respectively. Right bars indicate the additional sensory information carried by phase shifts combined with power features, with respect to sensory information carried by power alone. Stars indicate significantly positive quantities (*t* test between actual information values and bootstrapped information values, *p*-values Bonferroni-corrected). Data available at http://dx.doi.org/10.6084/m9.figshare.1460872.

**Fig 5:**
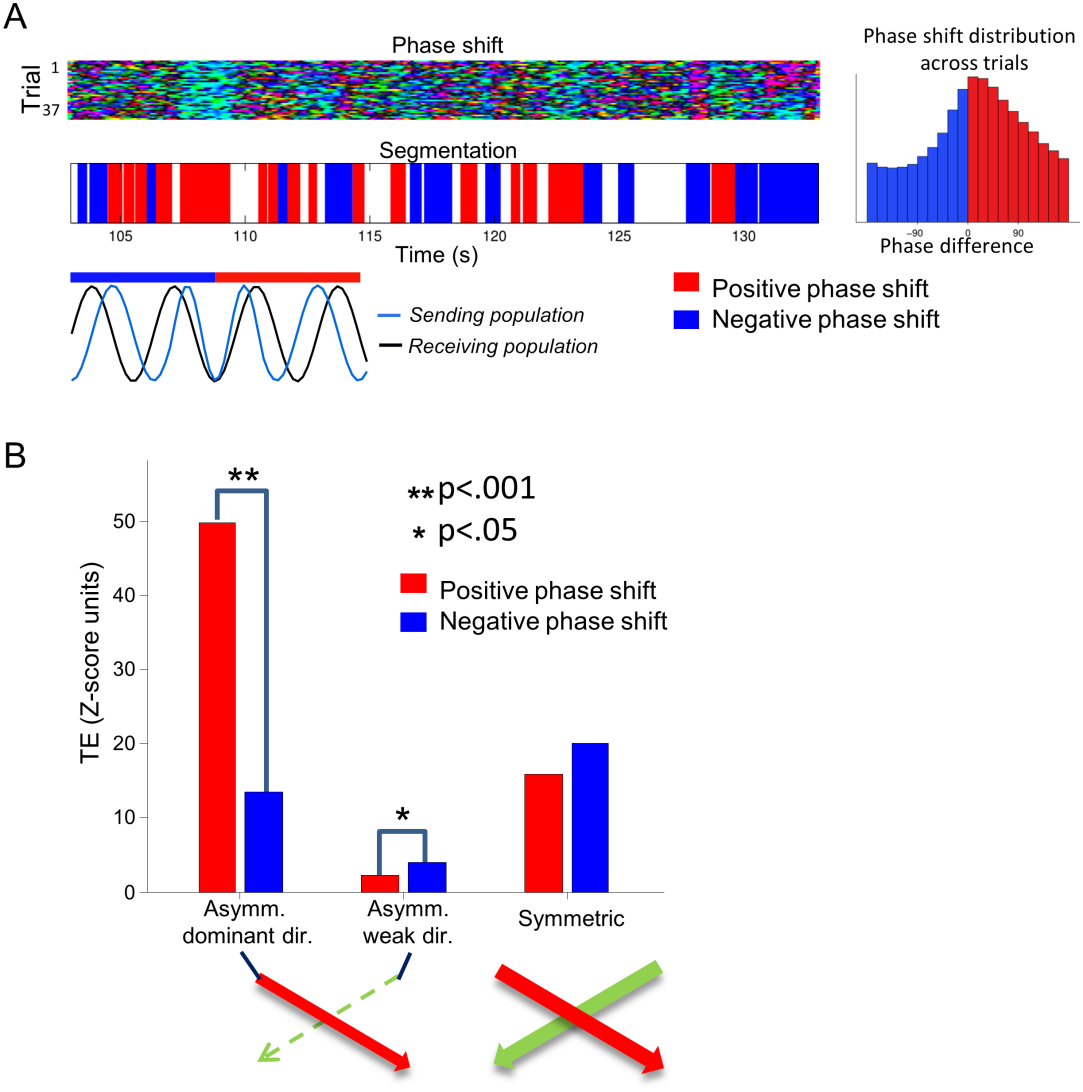
Changes in phase shift sign modulate dynamically directional information transfer. (A) Principle of the segmentation of the movie stimulation blocks illustrated on a pair of electrodes (the same one used in Fig 4A). For a given sending–receiving electrodes pair, the sign of the phase difference (whose meaning and color coding is illustrated in the schematic representation at the bottom of the panel) is first computed as a function of time for all trials. The histogram of phase differences across trials for this pair is represented on the right-hand side. Periods with a consistent dominant phase difference during a period superior to 300 ms are labelled according to the sign of the gamma phase difference. (B) TE values (Z-score units), from gamma phase to MUA, computed within blocks of positive and negative phase shifts, respectively. Results are presented as average over all relevant pairs across all sessions. Left: TE along the leading direction of causation (over the whole movie) for strongly asymmetric pairs. Middle: TE along the weakest direction of causation (over the whole movie) for strongly asymmetric pairs. Right: TE for symmetric electrode pairs. In this latter case, the average of TE over the two symmetric directions is shown. Stars indicate statistical differences (sign test). Data available at http://dx.doi.org/10.6084/m9.figshare.1460872.

An example of the time course over the movie presentation of the phase shift between two example electrodes in individual trials is shown in Fig 4A. To aid visualization, only 25 s of movie presentation were shown, and phase shifts were temporally smoothed by computing circular statistics over a 300 ms sliding window (we chose a window length of 300 ms because it is a time scale with high power of stimulus variations in these natural movies [32,34,50]). For a given window, circular mean of the phase shift was encoded in Fig 4A by the hue, while the consistency of the shift was quantified by the Phase Locking Value (PLV) and encoded by the intensity (see S1 Methods). Phase shifts were not constant in time, but varied in value and sign within each individual trial of the movie presentation (Figs 4A and 5A). Notably, there were specific time points (or scenes) during the movie presentation in which either positive or negative phase differences were elicited reliably across trials (see the red arrows in Fig 4A for examples of such periods), indicating that the dynamics of phase shifts is modulated by the properties of the visual stimulus.

To systematically quantify the relationship between phase shifts and visual stimuli, we computed the mutual information that phase shifts carry about which scene of the movie was being presented. Mutual information is a principled and comprehensive way to quantify whether the considered neural response varies reliably across trials from scene to scene (see Materials and Methods). In our experimental setting, it quantifies (in units of bits) how much the observation of a neural response reduces the uncertainty about which scene of the movie is shown.

The information about the movie scenes carried by phase shifts (averaged over all electrode pairs for each session) is shown in Fig 4B. Information in phase shifts was significant in all sessions (*t* test with False Discovery Rate control q = .05), meaning that phase shifts are indeed reliably modulated by the stimulus. In contrast, and consistently with previous reports [51], the gamma phase of each individual electrode carried very little information about the movie (Fig 4B). The fact that reliable modulation of phase shifts by the visual stimuli happens in absence of reliable modulations of the phase at each individual site means that the stimulus modulation of phase shifts cannot be explained by stimulus-evoked changes in the phase of individual sites. This suggests that stimulus-modulated spatial phase patterns reflect an emergent property of the relative dynamics and interactions between different cortical sites.

We further checked whether information in phase shifts could be explained by the stimulus modulation of the gamma-band power in each individual electrode [34,52]. Although the gamma power in each site carried significant stimulus information in this dataset (S2B Fig), the information in the joint observation of power at a given site and phase shifts with respect to another site was much higher in each session (paired *t* test and False Discovery Rate correction with q = .05) than information carried by power or by phase shifts alone (Fig 4B). This means that the gamma phase shifts and gamma power at each site are modulated by the stimulus in a largely complementary way. Similarly, we found (S2C Fig) that the information in gamma phase shifts was also complementary to that of the firing rate from the same electrode (note that under these stimulation and recording conditions, the local firing rate and gamma power are coupled quite tightly [34]). These findings imply that movie modulations of phase differences cannot be explained by modulations in local power or firing rate alone.

To understand how frequency specific were these phase modulations, we computed sensory information for phases and phase shifts in the lower frequency bands, 2–4 Hz, 5–15 Hz, 16–50 Hz (S2A Fig). Sensory information of phases in individual recording sites were on average larger or at least comparable to the information of phase shifts between sites at the same frequency, and were significant (*p* < .001, *t* test w.r.t. bootstrapped values; Bonferroni-corrected) in all sessions and frequencies, barring one single exception (phase at 15–50Hz for c08nm1). These results obtained for lower frequencies (<50 Hz) are in sharp contrast with results reported above for the gamma phase (Fig 4B). Thus, the emergence of stimulus-dependent phase shifts between sites in absence of a stimulus modulation of phases at individual recording sites is specific to gamma-band oscillations.

### Dynamic Changes in Phase Differences Correspond to Dynamic Changes in Causal Interactions

Results presented above showed the average phase differences computed over the entire period of movie stimulation strongly correlate with the dominant direction of interaction computed over the entire movie presentation. Concurrently, our mutual information analysis showed (Fig 4) phase differences between recording sites can vary reliably across different movie scenes. A natural question is whether such dynamic stimulus-induced changes in phase differences lead to dynamic changes in the strength of directed interactions between neural populations.

We addressed this question by studying, for each pair of electrodes, the relationship between changes over time of the phase shifts between the sites and the changes over time in causation (measured as TE) exerted by the gamma phase at one location to the spiking activity at another receiving location. We first individuated, for each pair of electrodes, blocks consisting of periods of dominant positive and negative phase shifts. The segmentation, illustrated in Fig 5A for an example pair of electrodes, was implemented as follows. We first computed the sign of phase difference for a given pair of electrodes at all time-points in each trial. Then we labelled time points with a majority of positive shifts across trials as positive phase shift points. Conversely, we labelled time points with a majority of negative phase shifts across trials as negative points. We then considered for further analysis only blocks made of continuous time segments with at least 300 ms of either entirely positive (“positive blocks”) or entirely negative (“negative blocks”) phase shift.

We first considered the strongly asymmetric pairs defined in our previous analysis over the entire period of movie presentation. For simplicity, in reporting the results of this analysis, for each pair we ordered the electrodes according to the dominant direction of TE computed across all the movie presentation time. The distribution of phase for such an electrode pair is plotted on the right-hand side of Fig 5A. As illustrated by this example, and as shown above (Fig 3C), almost all electrode pairs with strongly asymmetric causal interactions have an average phase lag aligned with the leading direction of causation, and thus had a positive average phase shift restricted to the 0–45° range. However, the time-varying phase shift in positive and negative blocks covered a broader range: across all recordings, 50% of the average phase shifts in a positive or negative block were contained between −20 to 105 degrees, suggesting that phase shift values in this range can modulate information transfer. For each electrode pair, we then computed TE between gamma phase at the sending location and spiking activity at the receiving location using only positive-phase or negative-phase blocks respectively. Due to their bias towards positive shifts, there was a larger total duration for positive than for negative phase blocks. To make the quantitative comparison of TE computed in this way as fair as possible, we randomly down-sampled the number of blocks such that approximately the same time length was used to compute TE in each condition. We then investigated whether the amount of TE (and thus the strength of causation) between gamma phase and spiking activity at a receiving location was stronger during blocks of positive phase.

We considered separately the modulation with the sign of the phase shift of TE in either the leading or the weaker direction of causation (Fig 5B). We found that the values of TE along the leading direction of causation were more than four times larger (*p* < .001; sign test) when the phase lags were positive (i.e., consistent with the gamma wave propagating along to the leading direction) than when the phase values were negative (i.e., consistent with the gamma wave propagating opposite to the leading direction). In contrast, we found that values of TE against the leading direction of causation were approximately twice larger (*p* < .05; sign test) when the phase lags were negative (i.e., consistent with a gamma wave propagating against the leading direction) than when the phase values were positive. In other words, when the phase shifts transiently point toward the direction of causation that is the leading one, on average over the experiment, then TE in the dominant direction is transiently enhanced and the one in the weaker direction is transiently suppressed. This effect is reversed when the phase shifts transiently point against the direction of causation that is the leading one on average.

All in all, these results suggest not only the time-averaged phase shift points toward the overall dominant direction of communication across an entire experiment, but that transient stimulus-related changes in phase shifts are accompanied by a relative increase in causation strength along the spatial direction indicated by the sign of the phase shift. In other words, transient changes in the direction of propagation of the putative travelling gamma waves correspond to transient relative increases of information transfer in the direction of putative wave propagation.

We finally studied the dynamic modulations of TE for electrode pairs that were classified above as having “symmetric interactions”, i.e., for pairs of electrodes classified as having comparable TE values in both directions (according to the criteria defined in previous section). For these symmetric electrode pairs, we found that the amount of causation was the same (*p* > .05; sign test) for both positive and negative phase shifts (Fig 5B). This suggests that for these pairs of sites their interactions remain “mutual” (i.e., symmetric) independently of transient changes of phase shifts.

### Relationship between Phase Shifts and Movie Features

**Fig 6:**
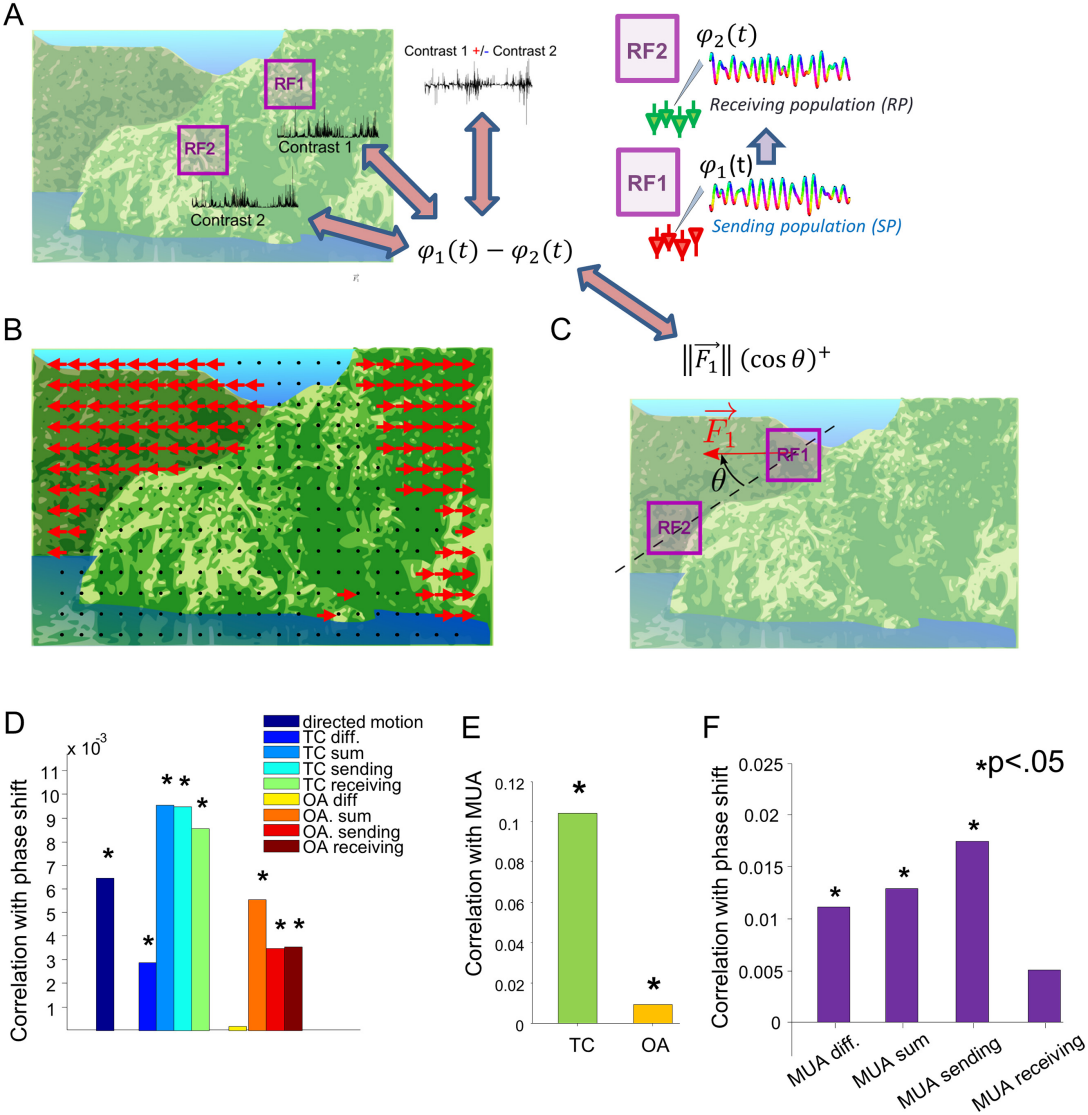
Relationship between gamma phase shifts and movie features within the RFs. (A) Principle of the computation of correlation between gamma phase shift of a strongly asymmetric channel pair and movie features (the local TC in this cartoon) measured in the RFs of each electrode in the pair. We extract the contrast time course from the movie data for each RF, symbolically represented over a movie frame, and check whether it is related to gamma phase shift time series for the corresponding electrode pair, by computing correlation across time between the two quantities. The same procedure is applied to the sum and the difference between features from the two RFs. (B) Example of optic flow estimation on one movie frame. Optic flow is estimated on a homogenous grid spreading over the frame. Nonvanishing optic flow estimates are indicated by a red arrow, and vanishing ones are indicated by a black dot. The schematic representation of the movie frame shows a sketch of the sky (light blue on top), the sea (dark blue at the bottom), an island (light green, center and right-hand side), and a remote landscape in the background (dark green, left-hand side). As the camera goes forward towards the island, the optic flow estimation correctly detects a movement of the landscapes towards the sides of the frame. (C) Principle of the computation of the correlation between gamma phase shift of an electrode pair and the directed motion along the inter-RF axis of this pair, derived from the optic flow vector ${\vec{F}}_{1}$ estimated in the sending recording site. The “+” superscript denotes the positive part. (D) Correlation across time between movie features (either a feature in an individual RF, sending or receiving, or the sum or difference of these features) and gamma phase shifts, averaged across asymmetric channel pairs. Local TC, OA, and directed motion are considered here as movie features. Stars indicate significant correlation values (signed rank test; *p* < .05). (E) Correlation across time between MUA activity and different movie features (TC or OA) in the RF, averaged across all electrodes. Stars indicate significant correlation values according to a signed rank test (*p* < .05). (F) Correlation across time of different variables derived from MUA activities of a channel pair (either a feature in an individual RF, or the sum of MUA in the sending and receiving RFs, or difference between MUA in the receiving and MUA in the sending RF) and the gamma phase shift of the pair, averaged across asymmetric electrode pairs. Stars indicate significant correlation values (signed rank test; *p* < .05). Data available at http://dx.doi.org/10.6084/m9.figshare.1460872.

The above analysis suggests that shifts in gamma phases, whose sign indicate the instantaneous direction of the gamma wave propagation and correlate with the TE, may modulate information transfer. A natural question is what kind of visual information about the stimulus is conveyed by these phase shifts. To address this question, we extracted various movie features estimated from the RFs of each channel (see S1 Methods), and then we correlated these movie features both with MUA firing rate in the same channel and with gamma phase shifts in the corresponding strongly asymmetric channel pairs. (see illustration in Fig 6A).

We first considered the tuning to the local TC in the RF. This was the RF visual feature that correlated the most with the MUA firing rate in the same site, with an increase in TC leading to an increase of MUA firing rate (Fig 6E). We found (Fig 6D) that both the TC at each individual site and its sum correlated positively with the phase shift in asymmetric pairs. Given that positive time shifts (i.e., time shifts along the overall prevalent direction of gamma wave propagation) lead to relative increases of TE in the overall dominant direction, this positive correlation between TC and phase shifts suggests that larger firing rate activation due to increase of TC (either in one of the RFs or in their sum) leads the gamma wave to travel from the sending to the receiving site and TE to increase in the direction of propagation. Given that the TC in the two RFs is partly correlated (S7 Fig), it is difficult to assess whether phase shifts are more modulated by the TC in one of the two RFs or by their sum. Interestingly, though, we found (Fig 6D) that the phase shift correlated positively with the difference between TC in the sending and the receiving site, which suggests that the direction of the travelling wave is influenced not only by the individual RF features but also by their combination (Fig 6D), with gamma waves more likely to travel from the sending site when the sending RF is more activated than the receiving one.

We repeated the analysis considering the OA in the RF, defined as the squared cosine of the difference between the orientation of the gradient in the RF and the preferred orientation of the corresponding channel. We found (Fig 6D) that OA modulated MUA firing rate and gamma phase shifts in a way similar to TC, although the correlation between OA and neural activity was overall much less strong than that of TC, and (unlike for TC) the correlation between difference of OA across RFs and phase shifts (though positive) did not reach statistical significance.

To gain insights into the overall effect on wave propagation of all the various visual features entering the movie, we computed the correlation between the trial-averaged MUA firing rate (a robust and general marker of the overall effectiveness of the visual stimulus drive at any given time point) and the phase shifts. We found (Fig 6F) that phase shifts correlated positively and significantly with the MUA in each RF, as well as with the sum and the difference between MUA in the sending and receiving RFs. The strongest correlation was found to be with the MUA in the sending RF. This, together with the positive correlation found with the difference between MUA in the sending and receiving RFs (Fig 6F), suggests that wave propagation from the sending RF and causation in the direction of the travelling wave is more likely when the sending RF contains one of its preferred features, and that the wave propagation is enhanced when the receiving RF is less activated by the stimulus. A simple interpretation of these results in terms of exchange of visual information is that the causal gamma waves propagate to communicate to nearby RFs the presence of their preferred feature, and that causation is particularly effective on the receiving site when the latter is not shown an optimal stimulus.

In addition to RF features mentioned above, we also computed a more global visual feature: the optic flow in the movie [53]. An example of estimated optic flow for one movie frame is given in Fig 6B. The optic flow in the movie generates an apparent movement in the visual field of the observer. The distributions of resulting speeds for all stimuli are presented in S8 Fig. The median values for the apparent speed were in the range 2.12–5.6 deg/s. Assuming the scaling of the parafoveal representation of the visual field in V1 is approximately 0.35 deg/mm, a purely feedforward mapping of object motion on the cortical tissue would result in propagating speeds in the range 6–16 mm/s. These values are much smaller than the speed values estimated in our analysis (approximately 360 mm/s). This suggests that optic flow generated by moving objects in the movie cannot generate patterns of gamma waves by simple feedforward entrainment to object motion. However, endogenously generated gamma waves might still play a role in the processing of this kind of motion. To test this hypothesis, we studied the relationship between the optic flow traversing the sending RF in the direction of the receiving RF, that we call “directed motion,” and the phase shift in asymmetric pairs (see illustration Fig 6B and S1 Methods). We found a positive correlation between the directed motion from the sending to the receiving RF and the gamma phase shift (Fig 6C). This shows that gamma wave propagation is modulated by extra-RF visual properties and corroborates the view that the travelling waves may communicate the presence of information that is salient to the sending RF.

## Discussion

To investigate how networks of neurons in V1 may exchange information, we analyzed the spatial structure of gamma-band LFP phase and its relationship to multiunit activity during presentations of naturalistic movies. These stimuli can trigger interactions between neural populations with both overlapping and nonoverlapping RFs and modulate these interactions over a range of perceptually relevant time scales. The relationship between phase of gamma oscillations and the effectiveness of neural communication has been previously investigated using spatially symmetric (nondirected) measures such as correlation and coherence [26]. Here we use asymmetric (direction specific) information theoretic tools to establish that—within V1—the local phase of gamma-band rhythmic activity exerts a dynamic, stimulus-modulated spatially-asymmetric directed effect on the firing rate of spatially separated populations. The spatiotemporal patterns of gamma phases can be compactly described as a stimulus-modulated putative gamma wave propagating along the prevalent spatial directions of communication. When the putative gamma wave transiently changed its direction (i.e., phase shifts changed sign), the relative amount of causation increased according to the direction of the propagating wave.

### Simultaneous Presence of Both Symmetric and Directed Communication in the Primary Visual Cortical Network

Gamma oscillations have been traditionally associated with symmetric interactions among neural populations, such as synchronization and coherence [23,54]. Such synchronized elements can naturally support important computations, such as tagging groups of neurons that participate in encoding of the same percept [17,55]. In our data, we found a proportion of site pairs exhibiting symmetric causal interactions accompanied by zero-lag synchronization between populations, supporting this view about the function of gamma oscillations.

However, we also found a more prominent proportion of recording pairs exhibiting a systematic nonzero lag gamma phase shift among spatially separate sites, that were accompanied by direction specific, rather than symmetric, communication between the sites. Our finding that these directed communications and the accompanying gamma phase shifts can be dynamically modulated by the stimulus suggests an important potential function for the phase relationships among oscillatory properties of different networks: the dynamic routing of the directional flow of information according to the needs of stimulus processing. This function cannot be easily accommodated by mutual zero-lag synchronization. While more theoretical work is needed to understand the importance and computational abilities of these dynamic directed interactions [56,57], it seems apparent that the simultaneous presence of functions such as dynamic one-directional routing and tagging of groups that process information together can only increase the range of sensory computations that can be implemented by the same anatomical network.

Another interesting question for future theoretical research is to study the conditions under which realistic network models can reproduce such a rich and heterogeneous dynamics with both symmetric and directional communication. It remains a theoretical challenge to understand how both phenomena may coexist within the same primary cortical network.

### Dynamic Changes in Spatial Gamma Phase Shift As Emergent Properties of the Intrinsic Interactions among Cortical Populations

An important finding is that gamma phase shifts between two sites can reliably change with the stimulus, and do so accompanied by simultaneous changes in the communication between the sites, in absence of a stimulus modulation of gamma phase at either site. This suggests that spatial gamma phase shifts reflect an emergent cooperative property of cortical dynamics that cannot be accounted for by feedforward sensory influences on individual sites. It is thus tempting to hypothesize that dynamic gamma phase shifts mediate dynamic stimulus- and direction-specific communication across cortical sites. A computational advantage of this putative mechanism of network reconfiguration is that it can control the cooperation among neuronal groups partly independently from the individual stimuli in the RF of each site, allowing flexible changes in neural communication depending on e.g., extra-RF sensory features and other contextual influences.

Our findings are indeed consistent with theoretical work suggesting that ongoing trajectories of network state reconfigurations participate in the mechanisms of processing of complex stimuli, such as those used in our experiment [58]. Our experimental observation moreover parallels the predictions of a recent modeling study [57], showing that modification of phase differences between interacting neural populations can lead to a rapid reconfiguration of their effective connectivity pattern.

### Frequency Specificity of Neural Interactions, Phase Patterns, and Sensory Information

Although we set out to investigate the specific hypothesis that directed network communication is modulated by gamma-band activity during sensory processing [26], we investigated a wide range of LFP frequencies. It is worth examining the implications of differences across frequencies in the results we found. TE causation values were significant for all bands, although the gamma-band had the largest causal effect during visual stimulation. Thus, our results support the notion that all bands can, in principle, be involved in information transfer. However, two key findings are specific to the gamma-band. First, a consistently significant and positive correlation between phase shifts and travelling waves (Fig 3B and S6 Fig), together with consistent stimulus-induced spatial coherence increases (Fig 1B) is found only for the gamma phase. Thus, the relationship between causation and stimulus-related travelling waves is strongly supported only for the gamma-band. Second, for the gamma-band, we can safely conclude that the propagating waves originate from neural dynamics rather than from spatiotemporal correlations in the movie, because the latter are too slow to account for the propagation of information by gamma waves. In particular, the same cannot be said about low frequency LFPs. Indeed, the spectral power of the movie features is highest in the low frequency range in which LFPs carry visual information [34]. Our previous modeling of visual cortex [59], as well as work on auditory cortex [60,61], suggest that the visual information in low frequency LFPs reflects the entrainment to the slow dynamics of natural stimuli, implying that low frequency waves may reflect stimulus dynamics rather than neural dynamics.

Great care should be taken when comparing the results obtained at different frequencies. On the one hand, the causal effect of lower frequency bands may be overemphasized with respect to that of the gamma-band, because lower frequency bands have often larger spatial coherence and likely capture the activity of neural populations of a larger size [31]. This caveat, while it does not affect the conclusion that gamma activity has the highest causal effect during visual stimulation, complicates the interpretation of the amount of causation across bands. On the other hand, a potentially larger spatial spread of lower frequency LFPs may penalize the ability to detect travelling waves at low frequencies, because it could compress the range of phase shifts attainable by low frequency oscillations (though we note that in our data, [2–4 Hz] phase shifts spanned a range similar to that of gamma phase shifts).

All in all, the caveats in comparisons across frequencies suggest that, although our results support the hypothesis of causal travelling waves in the gamma range, we cannot rule out that causal travelling waves might exist in other bands than gamma. Thus, while a long line of evidence links specifically gamma oscillations to the relative timing of interactions between local inhibitory and excitatory neurons [23,24,26,62–71], our results cannot support that the neural mechanism associated to the generation of gamma band oscillations are also exclusively responsible for the modulation of directed information flow.

### Putative Anatomical Pathways for Propagation of Gamma-Range Direction-Specific Interactions

The spatiotemporal pattern of gamma phases that we reported is consistent with the idea that gamma oscillations mediate direction-specific interactions by propagating along specific directions and over distances of several millimeters. Existence of functionally relevant travelling waves has been hypothesized in visual cortex [37,72]. An interesting question regards the possible anatomical substrate of this propagation of gamma-band activity. Our finding that sites with stronger causation tend to have similar orientation tuning and our estimation of propagation speed are compatible with the hypothesis that such interactions may travel along horizontal connections. The phase of gamma-band oscillations has been recently implicated in mechanisms for feedforward transmission of information across different areas in the visual cortical hierarchy [73,74]. Our results suggest that phase of gamma-band oscillations may also be involved in directional information transfers within a brain area, and that they may do so by modulating the propagation of information along lateral connections. Our data, however, do not speak on how this process may interact with top-down modulations of sensory processing from higher cortical areas; as such top-down contributions are likely to be minimal under the conditions of opiate anesthesia used for our data collection.

### Potential Confounds in Interpreting Directed Causal Effects of Gamma Phase on Spiking Activity of Spatially Separated Networks

We consider possible confounding factors in our measures of directed causal interactions between gamma phase and spiking activity at other sites. One possibility is that these relationships are not actually causal but they are rather due to cross-talk between signal at different locations or even to volume conduction. However, both the strong spatial asymmetry of the causal interactions that we observed and the finding that gamma-band interactions increase during stimulus presentation speak against such potential artifacts.

Another eventuality is that the causal effect of gamma phase may only show up artificially because firing rate and gamma phase of the sending population are correlated either because of locking of spike times to genuine network oscillations [23] or because of a spike-shape bleed-through on the LFP trace [75,76]. In other words, the reported causal effects of gamma phase may actually capture the effect of the level of firing rate of the sending population. The finding that LCI values are significantly positive (and thus that the same rate of the sending population may elicit a different firing rate in the receiving population depending on the gamma phase of the sending population), and the relatively small correlation between causation values exerted by spiking activity and gamma phase argue against this explanation. In addition, we note that we minimized spike bleed-through by removing the spike shapes prior to the LFP computation [76].

### Spatial Differences of Gamma Phases As Markers of Transient Direction-Specific Neural Interactions

An important practical consequence of our results is that phase shifts across multichannel recordings can be taken as meaningful markers for dynamic functional connectivity in distributed networks. Such phase relationships are easier to compute and much less data intensive than detailed measures of functional connectivity such as TE, and have been used as markers of interactions among areas [77–79]. The results we presented in this article elucidate some of the neural information transmission mechanisms that may be captured by observation of relationships between phases of the oscillatory activity of spatially separated neural populations, and provide a simple way to interpret the time-resolved sign of these measures in terms of directionality of dynamic information flow.

### Conclusion

Our study suggests that the dynamical relationships between gamma phases at different locations mark, and possibly causally mediate, the dynamic reconfiguration of functional network connections.

## Materials and Methods

### Ethical Statement

The data analyzed here was recorded as part of previous studies [34,40]. Recordings were obtained from the visual cortex of adult rhesus monkeys (Macaca mulatta) using procedures described below. All procedures were approved by local authorities (Regierungspräsidium Tübingen), were in full compliance with the guidelines of the European Community (EUVD 86/609/EEC) and were in concordance with the recommendations of the Weatherall report on the use of nonhuman primates in research.

### Electrophysiological Recordings and Data Processing 

Extracellular potentials were recorded in the V1 of three anesthetized monkeys for a total of four recording sessions (d04nm1, d04nm2, g97nm1, c98nm1), each performed on a different day. The experimental procedures and recording setup have been described elsewhere [34,40]. In each session, 6 to 11 tungsten electrodes were positioned according to a 4 x 4 square matrix (minimal interelectrode distance varied from 1 to 2.5 mm). During each session, between 30 and 120 trials of stimulation with color movies (duration ranging from 4 to 6.5 min) were recorded, as well as 5–10 trials of spontaneous activity (5 min duration). Spiking activity of neurons in the vicinity of each electrode was measured by extracting the MUA signal by high-pass filtering the extracellular potential above 1,000 Hz and subsequent rectification. The MUA signal obtained in this way measures the massed firing rate of a group of neuron in the vicinity of the electrode [30]. We used this measure of spiking activity because its values can be modeled by continuous random variables and are thus better suited to our information theoretic measures of spike field relationships than signals based on spike detection [40]. We extracted the LFP as follows. We cleaned the extracellular signal from spike bleed-through following the methodology proposed in [76] and code available at http://apps.mni.mcgill.ca/research/cpack/lfpcode.zip (we used multiunit spikes detected with a threshold of 3 SD for this purpose). We then down-sampled the extracellular signals (originally sampled at 20,835 Hz) to 1,000 Hz and low-pass filtered with a cutoff frequency of 100 Hz to obtain broad-band LFP. From this signal, we extracted band-specific LFPs by band-passing it using a zero lag 8th order Butterworth FIR filter in the specified frequency range (most analysis was done using the 50–80 Hz gamma-band). We extracted instantaneous phase and power of the considered oscillatory band using Hilbert transforms as detailed in S1 Methods.

### Sensory Information Calculations

To compute how different neurophysiological signals (such as phase or power at individual sites, or phase differences among sites) were modulated by the movie stimulus, we computed the Shannon Information (abbreviated as Information in this paper) between the set of stimuli *S* and the neural response *R*, defined as

I(S;R)=H(R)−H(R|S) (1)

where *H* stands for the Shannon entropy. The first term on the right hand side of Eq 1 is called the response entropy and quantifies the variability of the neural response *R* across all trials and stimuli, while the second term is called the noise entropy and quantifies the residual variability of *R* for a given stimulus *S*. Information thus measures the reduction of uncertainty on *R* when *S* is known [80]. To apply this approach to a complex, time-varying stimulus such as a naturalistic movie, following previous work [34,51,81], we divided the movie presentation time into nonoverlapping “scenes.” Scenes were 300 ms long except for individual phases, which are fast time-varying and thus studied using scenes of one oscillation period duration. We considered each such scene and the associated neural response as stimulus response-pairs in Eq 1. Thus, we computed how much information the neurophysiological response carried about which movie scene was presented. As such, it is a meaningful measure of how well and reliably the neural response is modulated during the movie. Information was computed by first binning the responses into a number of equipopulated bins and then applying statistical corrections to remove the limited sampling bias, using the Information Breakdown toolbox [82] available at http://www.sicode.eu/results/software.html (see S1 Methods for full details). All variables (except individual phases which vary at shorter time scales and were thus evaluated at a single point at the center of each scene, scene length having the duration of one period of oscillation) were smoothed using a 300 ms rectangular sliding time window and binned into four bins (we kept a low number of bins because of the low number of trials).

### TE between Extracellular Recordings 

TE is an information-theoretic measure of the causal dependency between the time series of a putative cause *X* and the time series of a putative effect *Y* in the framework of Wiener-Granger causality, stating that a signal *Y* is causing *X* if the knowledge of the past of *Y* reduces the uncertainty about the future of *X*. TE, along with other causal measures derived from the Wiener-Granger principle such as Granger Causality, is a widely used tool to infer causal functional connectivity from brain recordings. In many cases, the network inferred from these techniques matches well the patterns of anatomical connectivity [83] although the functional causal measures are also sensitive to the effect of dynamical variables such as the state of the network nodes that cannot be captured by anatomical connectivity [84]. The uncertainty of *X*_*t*_, the present value of *X*, is quantified by its entropy *H (X*_*t*_ *)*. Using this definition, TE compares the uncertainty of *X*_*t*_ given its past *X*_*past*_ with the uncertainty of *X*_*t*_ given both its past and the past of *Y*. The difference of these quantities defines the TE

$$T(Y\to X)=H(X_{t}|X_{past})-H(X_{t}|X_{past},Y_{past})$$ (2)

It can be shown [28,85] that the above equation corresponds to the mutual information between present activity of *Y* and past activity of *X*, conditioned on the past activity of *Y*:

$$T(Y\to X)=I(X_{t};Y_{past}|X_{past})$$ (3)

In our calculations, we almost always computed TE between the time series *Y* of the gamma-band phase at a putative sending location and the time series *X* of the spiking activity at a putative receiving location. Although, in one control analysis we used the spiking activity of the sending location as signal *Y*.

TE was computed by binning the responses into a number of equipopulated bins and then using statistical corrections to remove the limited sampling bias, using the Information Breakdown toolbox [82] available at http://www.sicode.eu/results/software.html. The routine for TE estimation itself is provided as supplementary material (http://dx.doi.org/10.6084/m9.figshare.1460872). Finally, TE values were Z scored to the bootstrapped values of TE that would be obtained in case of no causation between the time series only because of a common time history of sensory stimulation (See S1 Methods for details).

We note that TE is similar in concept to Granger causality [27] but has the additional advantage over Granger causality that (being based on mutual information) it captures all possible kinds of relationships between the variables.

### RF Estimation

We estimated the RF location using reverse correlation [86] between the gamma power in each channel and movie luminance. To ease computation, movie frames were spatially smoothed using an average over a 6 x 6 sliding window and then spatially down-sampled by a factor of 4. When using reverse correlation with natural stimuli, the obtained RF is likely to be larger than the true RF because of the spatial correlations in the stimulus [87,88]. To achieve better localization of RFs, we thus minimized large correlation between pixel luminance across the frames in some cases by removing the first one or two largest singular value decomposition (SVD) components of the spatiotemporal time course of the movie luminance. Correlation values were computed across time for each experiment between each pixel luminance and gamma power, using time series subsampled at 66Hz, and taking into account a time lag of 60ms between the stimulus and the neural response in V1, matching the order of magnitude reported in previous literature [88,89]. Correlation values were then Z-scored across experiments with the same movie stimulus, and the resulting maps for each movie were averaged together to get a final correlation map for each electrode in each recording session. An example of such correlation map is shown in S1B Fig. The RF center was chosen as the pixel achieving the maximum of this map, the RF shape was assumed square with vertical and horizontal borders, while the RF size was the smallest one achieving below 75% of the maximum value of the map on its border.

## Supporting Information

S1 Fig Summary of the properties of RFs and of the spatiotemporal structure of movie features. (A) Location of estimated RFs (colored boxes) for session d04nm1 relative to the area of the presented movie (delimited by the black border). (B) Reverse correlation of the stimulus with the resultant gamma power for a single channel. The estimated RF based on this map is also shown (purple rectangle). (C) Distribution of distances between RF centers for each electrode pair in visual angle, resolved by the causality category for the pair (see Results section “Information theoretic measures of the impact of gamma phase on neural activity at other locations”). (D) Distribution of the overlap of RFs for pairs of electrodes, shown for each causality category. (E) Temporal power spectral density of two movie features, local TC and OA, averaged over sessions, movies, and electrode RFs (see S1 Methods). (F) Scatter plot of the correlation across time of movie features between the two RFs associated with a pair of electrodes, against the distance between the centers of the RFs. This is resolved for different causality categories of electrode pairs.
(TIF)

S2 Fig Additional sensory information results. (A) Sensory information carried by phases and by phase shifts (averaged across all electrodes, and pairs of electrodes, respectively) for the 2–4 Hz, 5–15 Hz, and 16–50 Hz frequency bands. Results are reported separately for each session. Stars indicate significantly positive quantities (*t* test between actual information values and bootstrapped information values, with Bonferroni corrected *p*-values). (B) Sensory information computed for gamma power and MUA amplitude averaged across all electrodes and all sessions. Stars indicate significantly positive quantities according to a *t* test (Bonferroni corrected) between actual information values and bootstrapped information values. (C) Sensory information carried by several features of the gamma oscillations averaged across pairs of electrodes of each session. Left bars indicate the sensory information carried by gamma phase shifts, respectively. Right bars indicate the additional sensory information carried by phase shifts combined to MUA amplitude, with respect to sensory information carried by MUA amplitude alone. Stars indicate significantly positive quantities (*t* test between actual information values and bootstrapped information values, *p*-values Bonferroni corrected).
(TIF)

S3 Fig Spectral properties of LFP recordings. (A) LFP power spectra (averaged across all electrodes) for each of the four recording sessions (d04nm1, d04nm2, c98nm1, g97nm1). Grey lines show LFP spectra computed during spontaneous activity (i.e., when the screen used for visual stimulation was blank). Blue lines show spectra recorded in response to visual stimulation with Hollywood movie clips. Shaded areas indicate the SD across electrodes. Spectra were estimated using a Welch periodogram with 1 s duration blocks. (B) Ratio between the LFP power spectra obtained during movie stimulation and during spontaneous activity. Shaded areas indicate SD across electrodes.
(TIF)

S4 Fig Further analyses of the causal impact of gamma phase on neural activity at other locations. (A) Principle behind the calculation of TE, LCI, and localized LCI used to calculate the influence of gamma phase at a “sending” recording site on the spiking activity at a “receiving” site. We compute the information carried by a putative causal variable—the past gamma phase at the sending site—on the current spiking activity at the receiving site (green arrow), beyond the information due to the influence of the past of a conditioning variable, which is held constant (purple arrows). In the case of TE, the past spiking activity at the receiving site is conditioned on; for LCI, the conditioning variable is the past spiking activity at the sending site, whereas for loc. LCI it is the past phase of the receiving site instead. (B) LCI and loc. LCI values (Z-score units) averaged across all pairs of electrodes and sessions, during spontaneous activity and movie stimulation. Error bars indicate SE. Stars indicate significant increase during movie stimulation with respect to spontaneous activity (*t* test). (C) Histogram of LCI values across all pairs of electrodes in all sessions, cropped to the 95th percentile. The electrode pairs were divided into four equipopulated ranges of interelectrode distances.
(TIF)

S5 Fig Distribution of average phase shifts and interelectrode distance. (A) Scatter plot of the absolute value of the circular average (computed across movie time and trials) of gamma phase shift for each electrode pair plotted against interelectrode distance. Each marker corresponds to an individual electrode pair, and pairs are grouped by causality category: asymmetric pairs, symmetric pairs, and other (neither symmetric nor asymmetric) pairs. (B–D) Same as panel A for phase shift computed in different frequency bands (whose frequency range is reported in the top of the panel).
(TIF)

S6 Fig Correlation between phase shift and TE in different frequency bands. Spearman’s rank correlation (Bonferroni corrected) between the time-averaged phase shift for electrode pairs and the TE from phase to MUA of the same electrodes, computed separately for each session and relative to a specific LFP frequency band: 2–4 Hz, 5–15 Hz, 16–50 Hz, and 50–80 Hz.
(TIF)

S7 Fig Schematic of the computation of wave propagation speed. Estimation of the speed of gamma waves relies on estimating the spatial derivative of the phase of gamma oscillations. Spatial phase shifts are estimated along the direction of propagation of each strongly asymmetric pairs, taken as reference (see Supplemental Methods, section “Computation of the speed of wave propagation”). For such a pair, with causal relation shown in red, phase shifts are computed between the receiving electrode (red arrowhead) and electrodes along the direction of propagation (green nodes). The criteria for an electrode to be “on the direction of propagation” is that the displacement from this electrode to the reference receiving electrode (in blue), should be no more than 45° from the causal direction of the reference pair. We then measure the phase shift between the selected electrode and the reference-receiving electrode, and the corresponding distance travelled by the wave is the projection of the interelectrode distance of this pair onto the axis of the reference causal pair.
(TIF)

S8 Fig Angular speed of the optic flow in the movie stimuli. Distributions of angular speed values (pooled across all movie frames, sessions, and electrode RFs) of the optic flow estimated for each movie are represented by boxplots. The values are expressed in units of degrees of the animal’s visual angle per second (deg/s). For each box, the top and bottom are the 25th and 75th percentiles of the samples, respectively; the line in the middle of each box indicates the sample median; the dashed lines extending below and above each box are drawn from the ends of the interquartile ranges to the furthest observation (extreme points not considered as outliers); crosses (if any) in the diagrams indicate outliers. A data point is considered as an outlier whenever it is larger than Q3 + 1.5 * (Q3 − Q1) or smaller than Q1 − 1.5 * (Q3 − Q1), Q1 and Q3 indicating the 25th and 75th percentiles, respectively.
(TIF)

S1 Methods Supplemental methods and references. (PDF)

S1 Table Distribution of pairs with strongly asymmetric and symmetric interactions. The values reported indicate the proportion of electrode pairs of a given causality category (asymmetric or symmetric) that can be found at a given interelectrode distance range, with respect to the total number of pairs of this category, pooled across sessions.
(TIF)

## Abbreviations

LCI: Lagged Conditional Information
LFP: Local Field Potential
MUA: Multiple Unit Activity
OA: Orientation Activation
PLV: Phase Locking Value
RF: Receptive Field
SD: standard deviation
SE: standard error
TC: Time Contrast
TE: Transfer Entropy
V1: primary visual cortex
