# Supplementary figures and images for: Shifts of Gamma Phase across Primary Visual Cortical Sites Reflect Dynamic Stimulus-Modulated Information Transfer
Source: PLoS Biol. 2015 Sep 22;13(9):e1002257. doi: 10.1371/journal.pbio.1002257 (PMC4579086; doi:10.1371/journal.pbio.1002257)

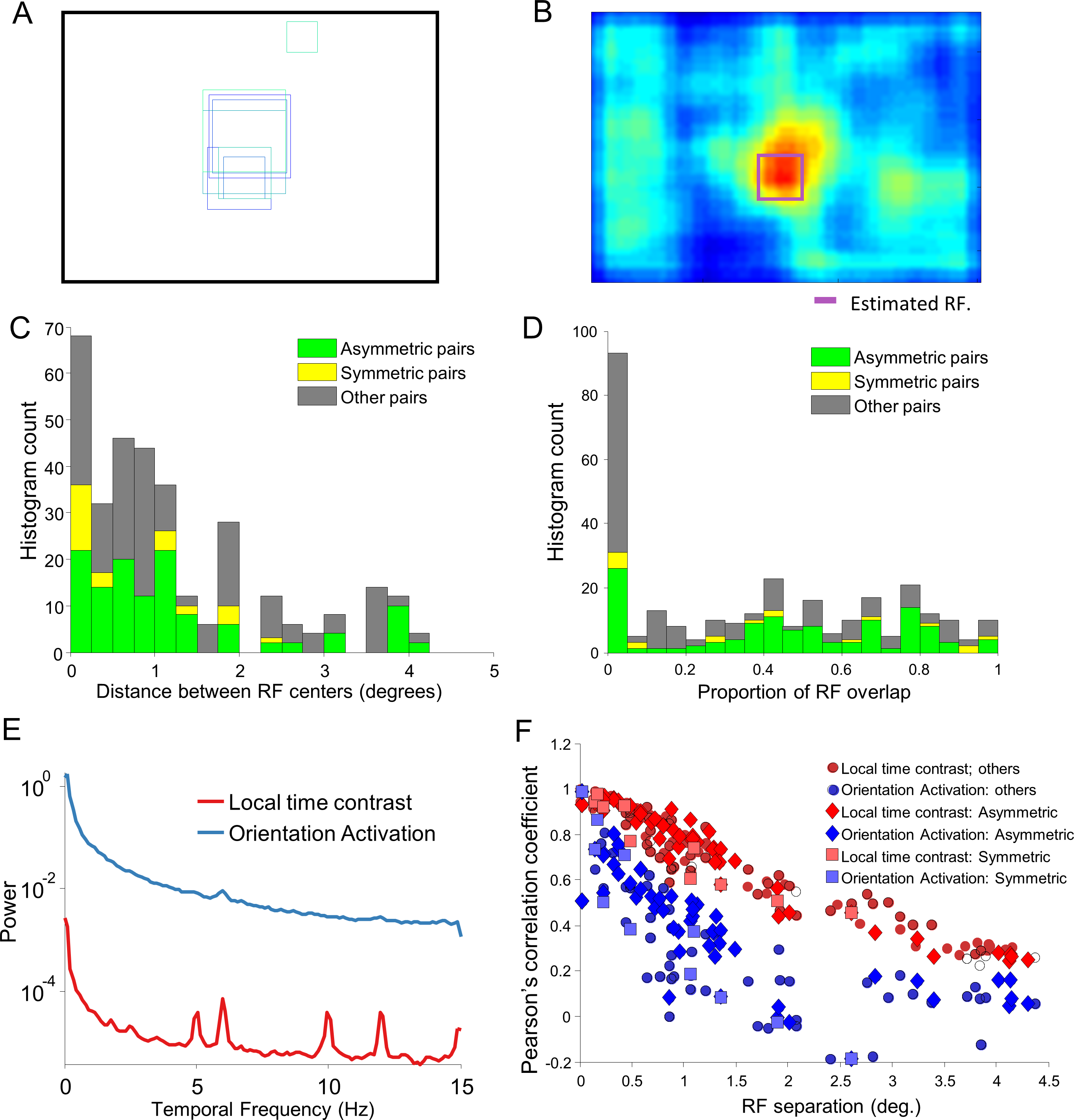

Supplement: S1 Fig — (A) Location of estimated RFs (colored boxes) for session d04nm1 relative to the area of the presented movie (delimited by the black border). (B) Reverse correlation of the stimulus with the resultant gamma power for a single channel. The estimated RF based on this map is also shown (purple rectangle). (C) Distribution of distances between RF centers for each electrode pair in visual angle, resolved by the causality category for the pair (see Results section “Information theoretic measures of the impact of gamma phase on neural activity at other locations”). (D) Distribution of the overlap of RFs for pairs of electrodes, shown for each causality category. (E) Temporal power spectral density of two movie features, local TC and OA, averaged over sessions, movies, and electrode RFs (see S1 Methods). (F) Scatter plot of the correlation across time of movie features between the two RFs associated with a pair of electrodes, against the distance between the centers of the RFs. This is resolved for different causality categories of electrode pairs. (TIF) [file pbio.1002257.s001.tif]

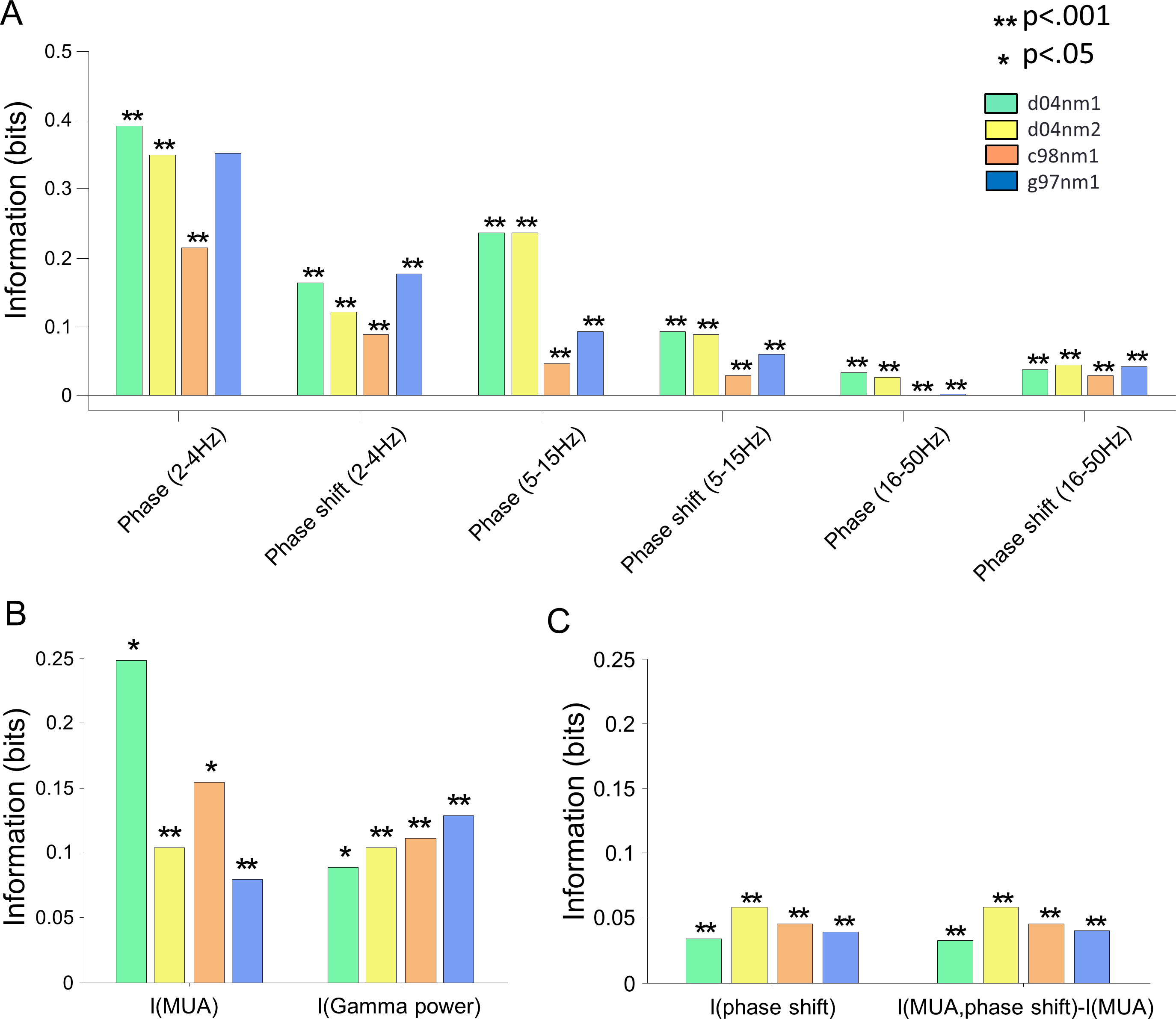

Supplement: S2 Fig — (A) Sensory information carried by phases and by phase shifts (averaged across all electrodes, and pairs of electrodes, respectively) for the 2–4 Hz, 5–15 Hz, and 16–50 Hz frequency bands. Results are reported separately for each session. Stars indicate significantly positive quantities (t test between actual information values and bootstrapped information values, with Bonferroni corrected p-values). (B) Sensory information computed for gamma power and MUA amplitude averaged across all electrodes and all sessions. Stars indicate significantly positive quantities according to a t test (Bonferroni corrected) between actual information values and bootstrapped information values. (C) Sensory information carried by several features of the gamma oscillations averaged across pairs of electrodes of each session. Left bars indicate the sensory information carried by gamma phase shifts, respectively. Right bars indicate the additional sensory information carried by phase shifts combined to MUA amplitude, with respect to sensory information carried by MUA amplitude alone. Stars indicate significantly positive quantities (t test between actual information values and bootstrapped information values, p-values Bonferroni corrected). (TIF) [file pbio.1002257.s002.tif]

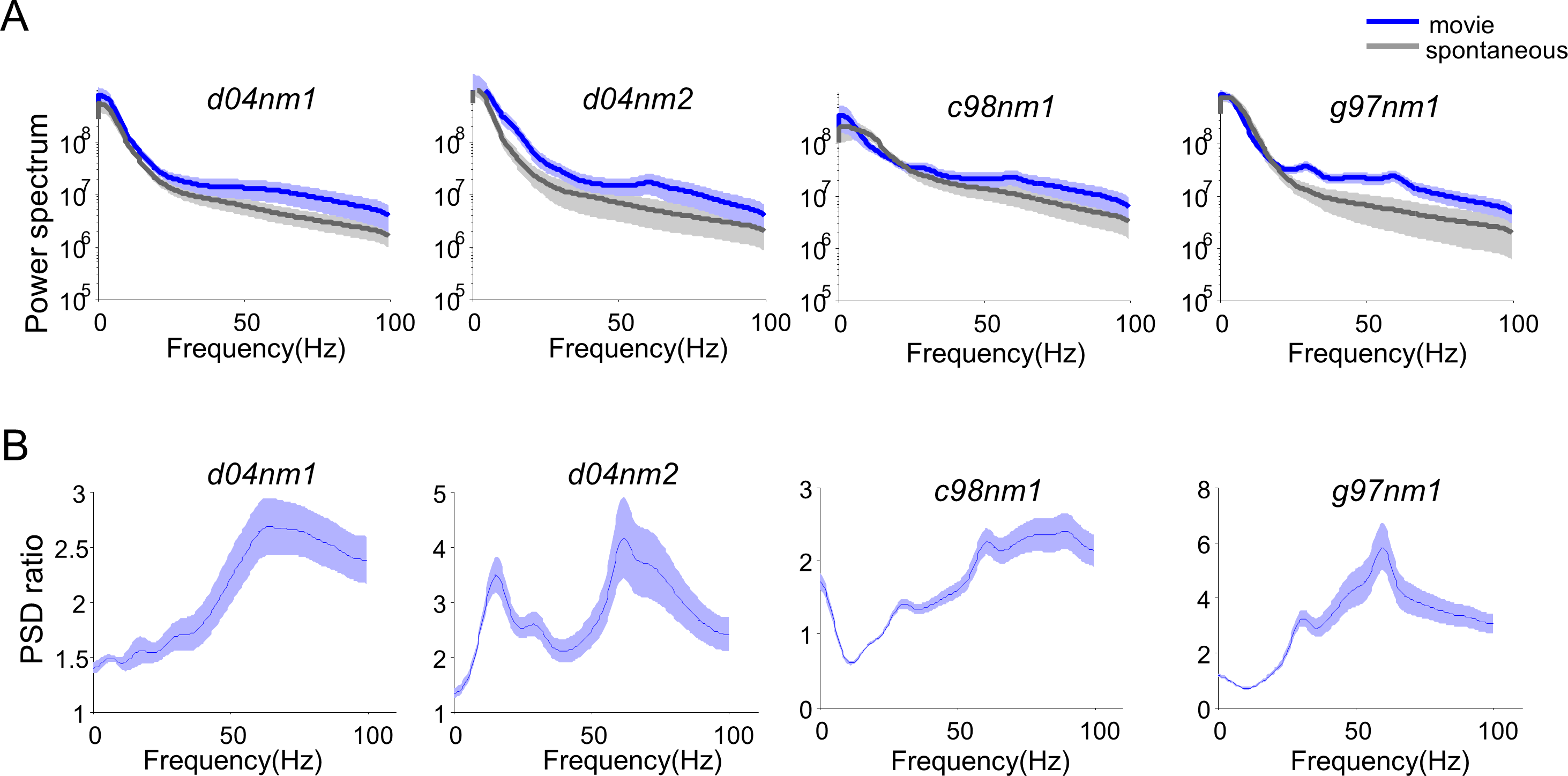

Supplement: S3 Fig — (A) LFP power spectra (averaged across all electrodes) for each of the four recording sessions (d04nm1, d04nm2, c98nm1, g97nm1). Grey lines show LFP spectra computed during spontaneous activity (i.e., when the screen used for visual stimulation was blank). Blue lines show spectra recorded in response to visual stimulation with Hollywood movie clips. Shaded areas indicate the SD across electrodes. Spectra were estimated using a Welch periodogram with 1 s duration blocks. (B) Ratio between the LFP power spectra obtained during movie stimulation and during spontaneous activity. Shaded areas indicate SD across electrodes. (TIF) [file pbio.1002257.s003.tif]

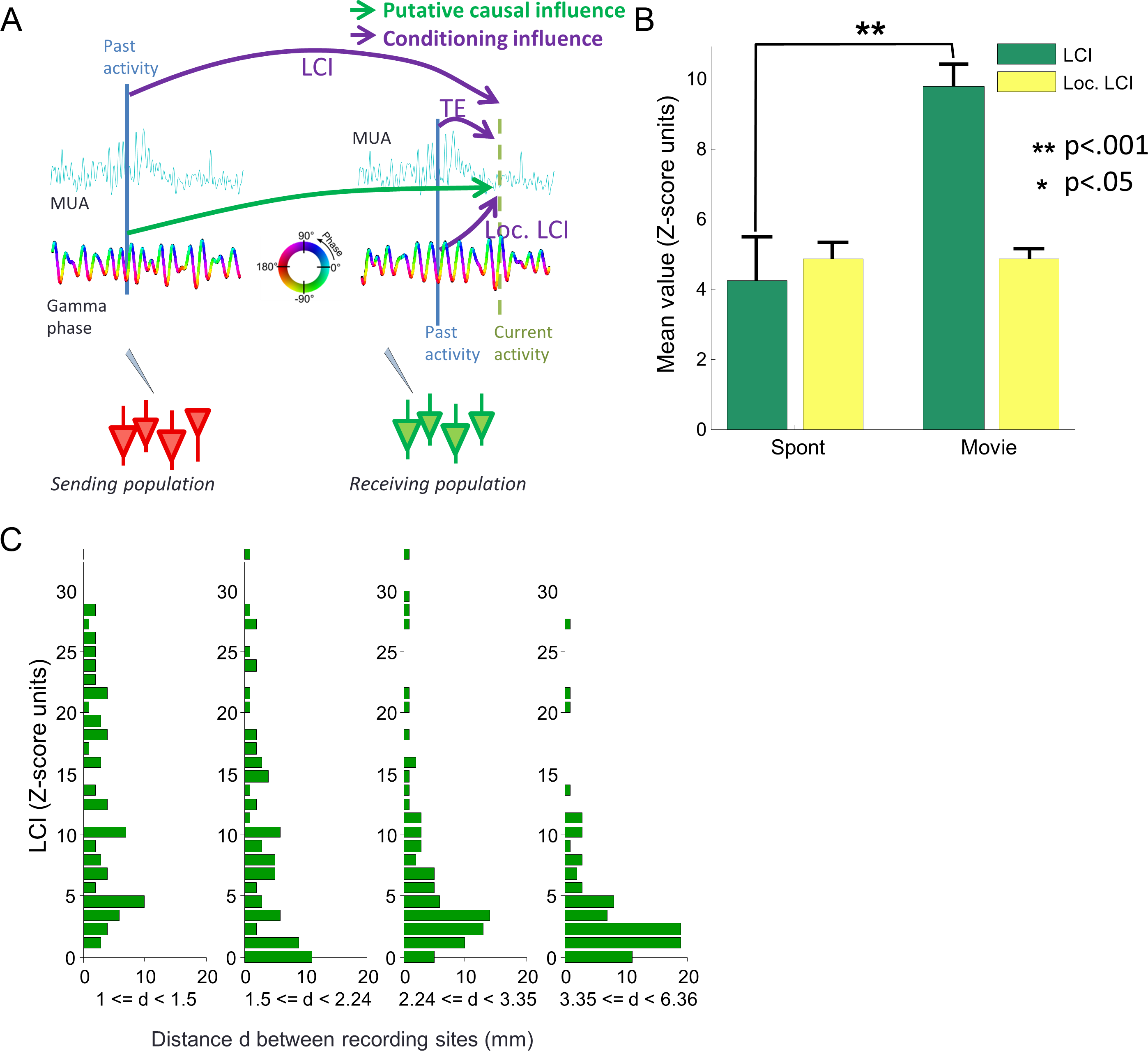

Supplement: S4 Fig — (A) Principle behind the calculation of TE, LCI, and localized LCI used to calculate the influence of gamma phase at a “sending” recording site on the spiking activity at a “receiving” site. We compute the information carried by a putative causal variable—the past gamma phase at the sending site—on the current spiking activity at the receiving site (green arrow), beyond the information due to the influence of the past of a conditioning variable, which is held constant (purple arrows). In the case of TE, the past spiking activity at the receiving site is conditioned on; for LCI, the conditioning variable is the past spiking activity at the sending site, whereas for loc. LCI it is the past phase of the receiving site instead. (B) LCI and loc. LCI values (Z-score units) averaged across all pairs of electrodes and sessions, during spontaneous activity and movie stimulation. Error bars indicate SE. Stars indicate significant increase during movie stimulation with respect to spontaneous activity (t test). (C) Histogram of LCI values across all pairs of electrodes in all sessions, cropped to the 95th percentile. The electrode pairs were divided into four equipopulated ranges of interelectrode distances. (TIF) [file pbio.1002257.s004.tif]

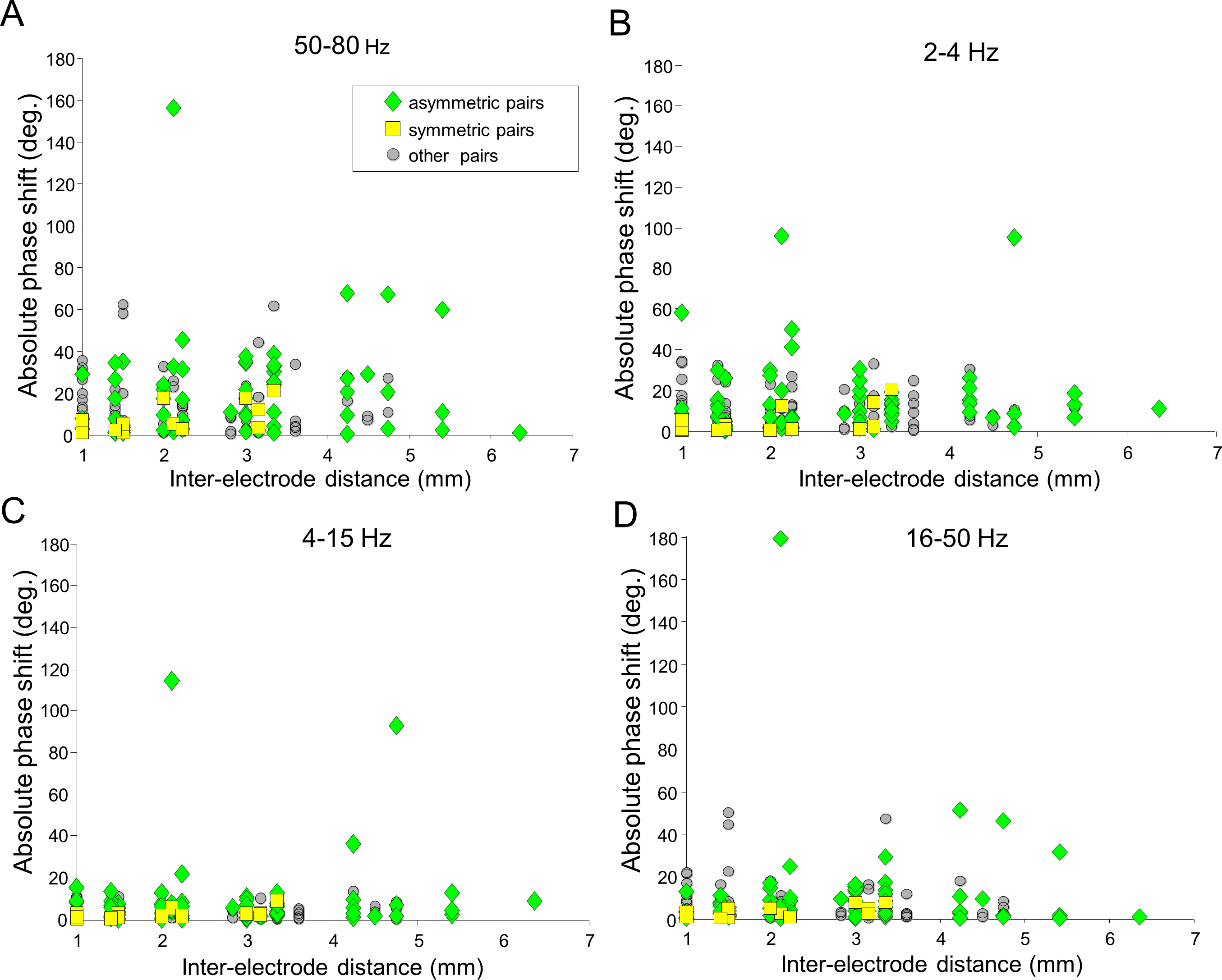

Supplement: S5 Fig — (A) Scatter plot of the absolute value of the circular average (computed across movie time and trials) of gamma phase shift for each electrode pair plotted against interelectrode distance. Each marker corresponds to an individual electrode pair, and pairs are grouped by causality category: asymmetric pairs, symmetric pairs, and other (neither symmetric nor asymmetric) pairs. (B–D) Same as panel A for phase shift computed in different frequency bands (whose frequency range is reported in the top of the panel). (TIF) [file pbio.1002257.s005.tif]

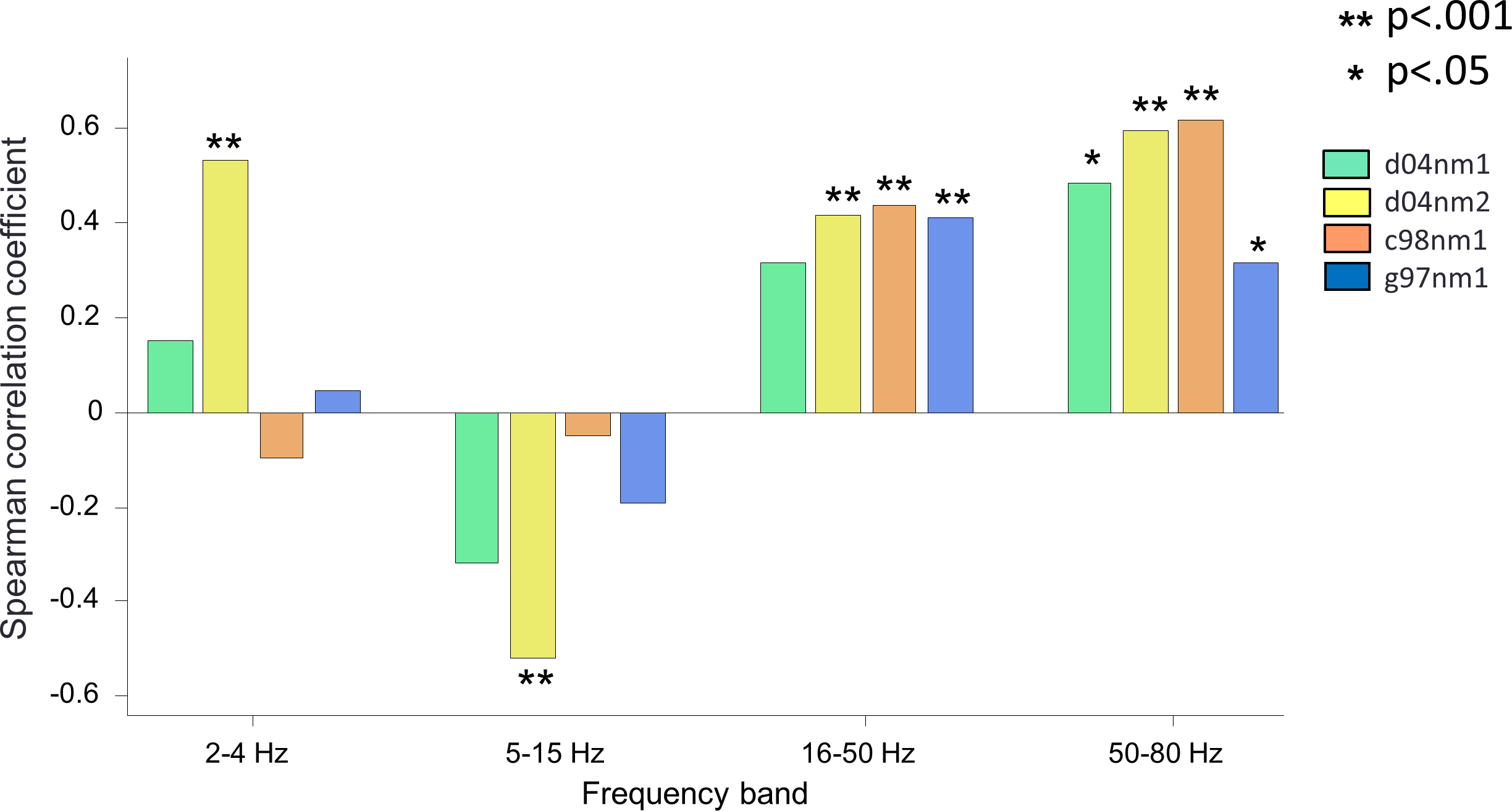

Supplement: S6 Fig — Spearman’s rank correlation (Bonferroni corrected) between the time-averaged phase shift for electrode pairs and the TE from phase to MUA of the same electrodes, computed separately for each session and relative to a specific LFP frequency band: 2–4 Hz, 5–15 Hz, 16–50 Hz, and 50–80 Hz. (TIF) [file pbio.1002257.s006.tif]

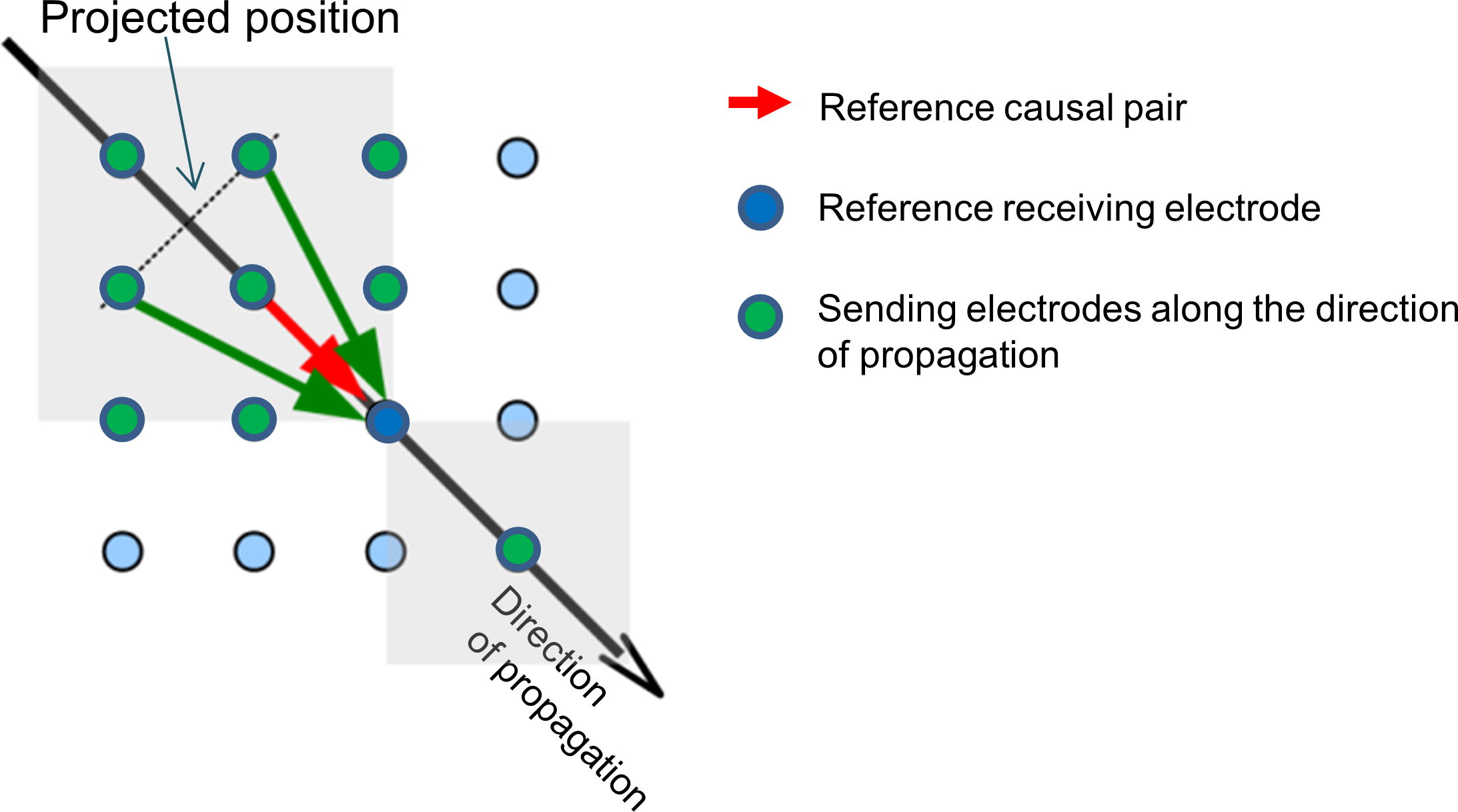

Supplement: S7 Fig — Estimation of the speed of gamma waves relies on estimating the spatial derivative of the phase of gamma oscillations. Spatial phase shifts are estimated along the direction of propagation of each strongly asymmetric pairs, taken as reference (see Supplemental Methods, section “Computation of the speed of wave propagation”). For such a pair, with causal relation shown in red, phase shifts are computed between the receiving electrode (red arrowhead) and electrodes along the direction of propagation (green nodes). The criteria for an electrode to be “on the direction of propagation” is that the displacement from this electrode to the reference receiving electrode (in blue), should be no more than 45° from the causal direction of the reference pair. We then measure the phase shift between the selected electrode and the reference-receiving electrode, and the corresponding distance travelled by the wave is the projection of the interelectrode distance of this pair onto the axis of the reference causal pair. (TIF) [file pbio.1002257.s007.tif]

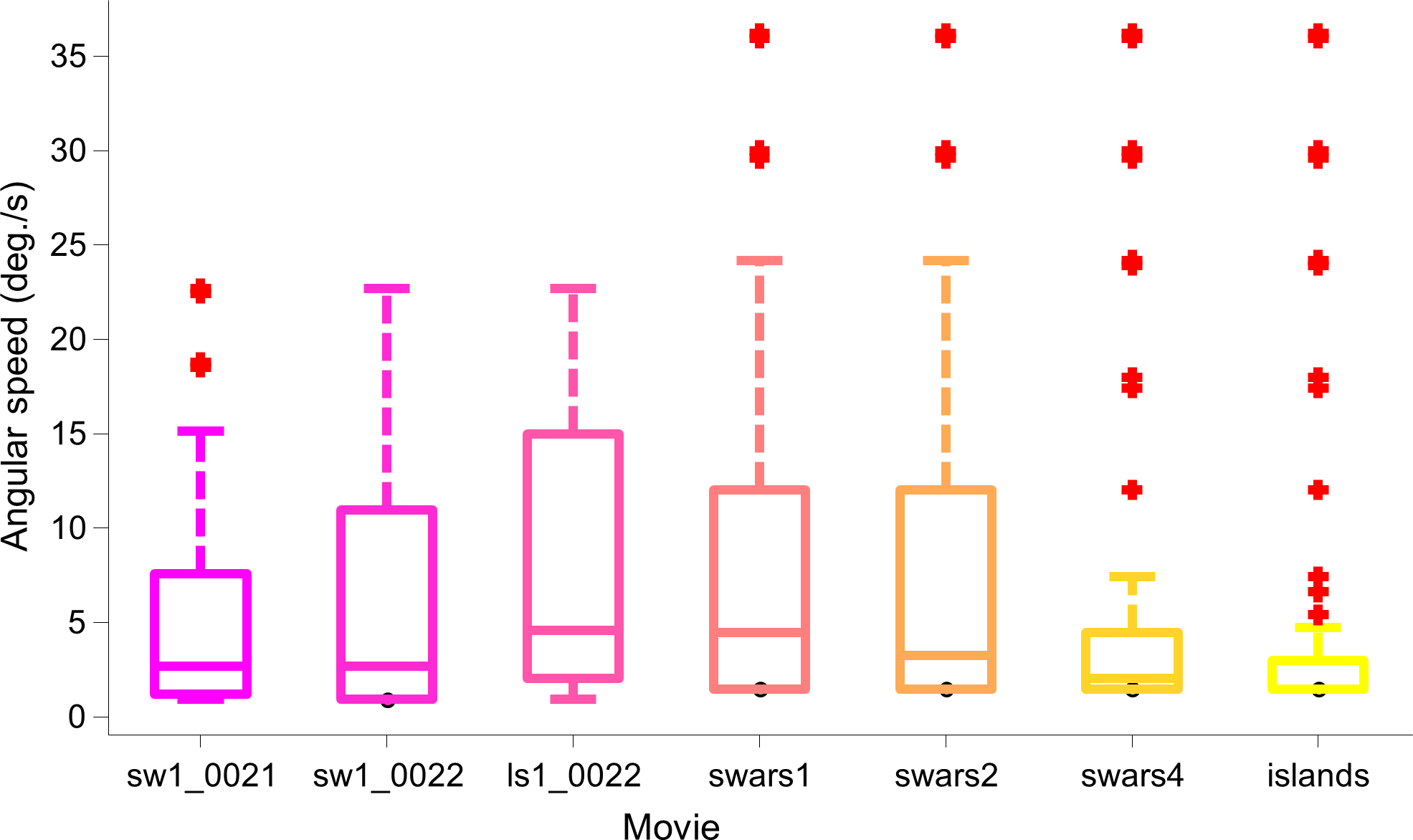

Supplement: S8 Fig — Distributions of angular speed values (pooled across all movie frames, sessions, and electrode RFs) of the optic flow estimated for each movie are represented by boxplots. The values are expressed in units of degrees of the animal’s visual angle per second (deg/s). For each box, the top and bottom are the 25th and 75th percentiles of the samples, respectively; the line in the middle of each box indicates the sample median; the dashed lines extending below and above each box are drawn from the ends of the interquartile ranges to the furthest observation (extreme points not considered as outliers); crosses (if any) in the diagrams indicate outliers. A data point is considered as an outlier whenever it is larger than Q3 + 1.5 * (Q3 − Q1) or smaller than Q1 − 1.5 * (Q3 − Q1), Q1 and Q3 indicating the 25th and 75th percentiles, respectively. (TIF) [file pbio.1002257.s008.tif]

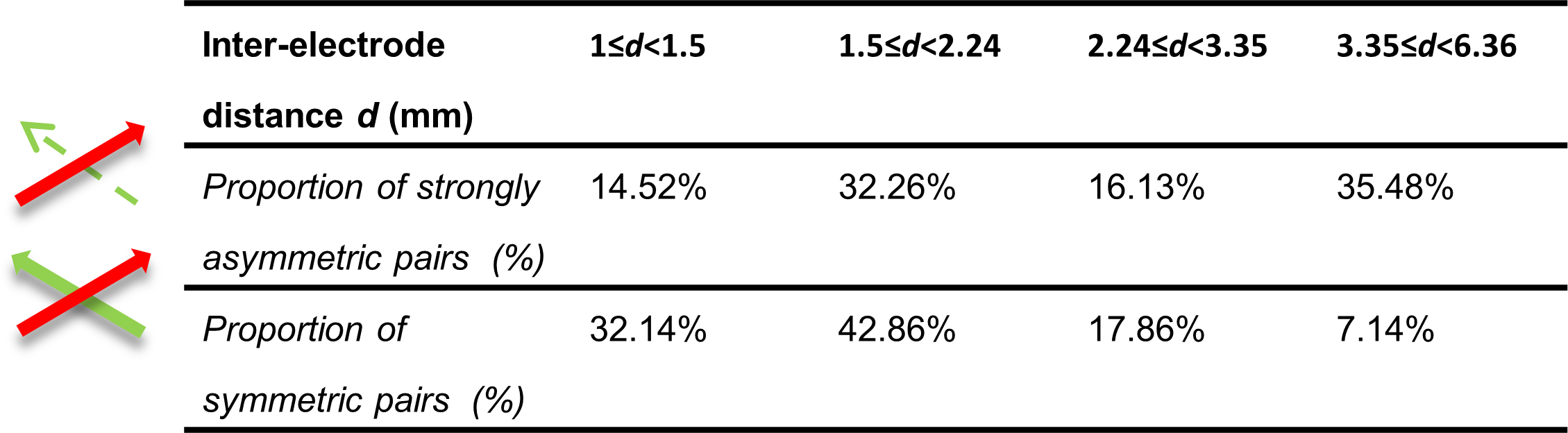

Supplement: S1 Table — The values reported indicate the proportion of electrode pairs of a given causality category (asymmetric or symmetric) that can be found at a given interelectrode distance range, with respect to the total number of pairs of this category, pooled across sessions. (TIF) [file pbio.1002257.s010.tif]
