# Supplementary material for: Shifts of Gamma Phase across Primary Visual Cortical Sites Reflect Dynamic Stimulus-Modulated Information Transfer
Source: PLoS Biol. 2015 Sep 22;13(9):e1002257. doi: 10.1371/journal.pbio.1002257 (PMC4579086; doi:10.1371/journal.pbio.1002257)
Supplement: S1 Methods — (PDF) [file pbio.1002257.s009.pdf]

Supplemental Methods

Shifts of gamma phases across primary visual cortical sites reflect dynamic stimulus-modulated information transfer

Michel Besserve[1,2]*, Scott C. Lowe[3], Nikos K. Logothetis[1,4], Bernhard Schölkopf[2] and Stefano Panzeri[5]

1. Max Planck Institute for Biological Cybernetics, Spemannstr. 38, 72076 Tübingen, Germany.
2. Max Planck Institute for Intelligent Systems, Spemannstr. 38, 72076 Tübingen, Germany.
3. Institute for Adaptive and Neural Computation, School of Informatics, University of Edinburgh, Edinburgh, EH8 9AB, UK.
4. Division of Imaging Science and Biomedical Engineering, University of Manchester, Manchester M13 9PT, United Kingdom.
5. Laboratory of Neural Computation, Center for Neuroscience and Cognitive Systems @UniTn, Istituto Italiano di Tecnologia, Via Bettini 31, 38068 Rovereto, Italy.

* michel.besserve@tuebingen.mpg.de

Contents

Supplemental Methods 2
- INSTANTANEOUS PHASE AND POWER ANALYSIS 2
- INFORMATION THEORETIC ANALYSIS 2
  - Lagged Conditional mutual Information (LCI) 2
  - Practical details of computation of Sensory Information from neural data 3
  - Practical details of Computation of Transfer Entropy and Lagged Conditional mutual Information from neural data 3
  - Spatial asymmetry index of the information transfer measures 4
- COMPUTATION OF THE SPEED OF WAVE PROPAGATION 4
- ANALYSIS OF VISUAL FEATURES 5
  - Orientation tuning curves 5
  - Optic flow estimation 5
  - Movie features estimation 5
  - Correlation between movie features and phase shift 5

Supplemental References 6

Supplemental Methods

INSTANTANEOUS PHASE AND POWER ANALYSIS

To quantify the relationship between phases of gamma oscillations, we rely on the use of the Hilbert transform and circular statistics. The Hilbert transform is a filtering technique that enables us to compute a complex signal, called analytic signal and defined as

$$x_a(t) = a(t)\exp(i2\pi\varphi(t)), \quad \text{(S1)}$$

where $a(t)$ is the instantaneous amplitude of the original signal $x(t)$ and $\varphi(t)$ is the instantaneous phase, such that the original signal is composed

$$x(t) = a(t)\cos(2\pi\varphi(t)). \quad \text{(S2)}$$

As a consequence, the instantaneous phase is the argument of the complex number $x_a(t)$ and we use the normalized Hilbert transform

$$\frac{x_a(t)}{a(t)} = \exp(i\varphi(t)) \quad \text{(S3)}$$

to study its statistical properties. Moreover, the instantaneous power in this frequency band is given by the squared instantaneous amplitude $a^2(t)$.

If we are given a set of time points $\{t_k\}$, the mean resultant vector

$$\vec{v} = \frac{1}{N}\sum_{k=1}^{N} e^{i\varphi(t_k)} \quad \text{(S4)}$$

is used to characterize the distribution of phases at these times. If phases are distributed around a mean phase with only a small spread, the mean resultant vector sums vector pointing in similar directions, resulting in a vector of large length (close to 1) with the same direction. Conversely, if phases are completely random, the mean resultant vector will have a very small length. The length of $\vec{v}$, which we call the Phase Locking Value (PLV) is the statistic used to test non-uniformity of the phase in the Rayleigh test [1]. The argument of $\vec{v}$ is called the circular mean and is used in our analysis to estimate the average phase or phase shift over a time interval.

INFORMATION THEORETIC ANALYSIS

Lagged Conditional mutual Information (LCI)

Definition

To quantify the effect of the gamma phase at the putative sending location on the spiking activity at the receiving location, we also computed a quantity that we called the Lagged Conditional Information (LCI) and that was defined as the information between present spiking activity at the receiving location and past gamma phase at the sending location, conditioned on the past spiking activity at the sending location. This can be expressed as:

$$LCI(Y_{sending} \to X_{receiving}) = I(X_{t,receiving}; Y_{past,sending} \mid X_{past,sending}), \quad \text{(S5)}$$

where X and Y represent spiking activity and gamma phase respectively, and the location at which these quantities are measured is indicated in subscript. Conditioning upon the past activity of the sending electrode insures that positive LCI values are interpreted as evidence that the relationship between gamma phase of the sending population and the spiking activity of the receiving population cannot be accounted for by the relationship between spiking activity and gamma phase at the sending location. As a further control, we also computed a Local Lagged Conditional Information (loc. LCI) by instead conditioning on the past phase at the receiving location, which is thus:

$$loc.\,LCI(Y_{sending} \to X_{receiving}) = I(X_{t,receiving}; Y_{past,sending} \mid Y_{past,receiving}) \quad \text{(S6)}$$

using the same conventions as in Equation (S5).

Results

The results of the TE analysis are reported extensively in the main text. In the following we summarize briefly the results of the LCI and loc. LCI analysis. The population average of LCI and loc. LCI values (Figure S4B) - which were also computed by Z-scoring them with respect to the distribution of spurious information values - were also highly significant (more than 8 in Z score units for LCI, more than 4 in Z-score units for loc. LCI; $p < 10^{-3}$, t-test). These values were significant at the individual electrode pair level for 95% of electrode pairs in both cases (t-test; False Discovery Rate control with q=.05). A simple way to interpret these positive LCI, loc. LCI and TE values is that the gamma phase in the sending population elicits a specific causal effect on the firing rate of the receiving population that cannot be explained by other variables such as the firing rate at the sending site or the phase of gamma oscillations at the receiving site at previous times. Moreover, there was a more than two-fold highly significant (p<.001; t-test across pooled electrode pairs of all sessions) increase in LCI magnitude during movie stimulation with respect to spontaneous activity (Fig S4). On the contrary, no significant increase in loc. LCI was found during visual stimulation.

We also investigated how LCI depends on the distance between recording sites. Fig S2C reports the histogram of LCI values of the pairs of electrodes across all sessions. The data were partitioned into four equipopulated ranges of inter-electrode distances. While a larger number of high LCI values could be observed at short distances (<2.24mm), several pairs with large inter-electrode distances also exhibited large interactions. In addition, electrode pairs exhibiting very low values could be found at all distances, supporting that LCI is not a simple function of distance and arguing against that they could be ascribed to external artifacts or volume conduction.

Practical details of computation of Sensory Information from neural data

The methodology for sensory information estimation, previously used in [2-4], relies on the estimation of the mutual information between the stimuli and features of the neural response R according to the formula:

$$I(S;R) = H(R) - H(R \mid S) \tag{S7}$$

Where the response entropy and the noise entropy are defined as follows:

$$\begin{aligned} H(R) &= -\sum\nolimits_{r} P(r)\log_2\left(P(r)\right) \\ H(R \mid S) &= -\sum\nolimits_{r,s} P(s)P(r \mid s)\log_2\left(P(r \mid s)\right) \end{aligned} \tag{S8}$$

In the above equations, P(s) is the probability of showing the stimulus s, P(r) the probability of measuring the response r, and P(r|s) is the probability of observing response r when the input is s. In our analysis, s corresponds to a given frame of the movie stimulus, and the probability distribution P(s) is uniform among all movie frames. To allow an efficient estimation of the sensory information, we binned each neurophysiological response into 4 equipopulated bins. To correct for the upward bias due to limited sampling, we first used the Panzeri-Treves correction [5] to estimate and subtract the bias, and we then removed any residual bias by further subtracting information values obtained by randomly permuting stimuli *s* and response features in each trial [6]. Finally, the significance of the Information values were tested by comparing the actual value to the bootstrap distribution obtained by pairing at random sensory inputs to feature values [7].

Practical details of Computation of Transfer Entropy and Lagged Conditional mutual Information from neural data

The Lagged Conditional mutual Information (LCI) and Transfer Entropy (TE) were estimated between time series *X* and *Y* using mutual information estimation techniques. In the following *X* and *Y* denote the time series of the spiking activity at the receiving electrode and the gamma phase at the sending electrode respectively. Here we just describe the procedure for computing TE, pointing out at the end of the section what the difference would be for LCI.

A problem in estimating TE directly from Eq. 2 of main text is the difficulty – for sampling reasons - to condition on the entire past of the variables. Therefore it is customary [8] to simplify the calculation by considering the signals at only one time point in the past, chosen with a delay parameter τ , as follows:

$$T(Y \rightarrow X) = (H(X_t \mid X_{t-\tau}) - H(X_t \mid X_{t-\tau}Y_{t-\tau})) \tag{S9}$$

The parameter τ is the delay at which we evaluate the past values of the time series. These TE values were computed for values of τ ranging from 1ms to 13ms (one gamma oscillation per period) and the

delay for which the average normalized TE across all pairs of electrodes and across all sessions was maximal was used for subsequent analysis [9]. We used equipopulated binning (with a value of 5 bins) of the time series to estimate the joint probability distribution of the variables $X_t, X_{t-\tau}, Y_{t-\tau}$. In addition, time points were decimated by a factor of 5 to limit the bias of TE values due to signal autocorrelation. The sampling bias of the TE computed from the discretized empirical probabilities was then corrected as described above for mutual information and as further detailed in [9]. As any causality measure, TE can be affected by confounders which would influence both signals *X* and *Y*. In our case, it is possible that the causal measure is influenced by the driving of both signals by the sensory input. To compensate for this effect, we estimated the amount of TE resulting from this common driving by computing TE between signals originating from different experiments with the same movie stimulus. Since these signals were not recorded at the same time, Transfer Entropy computed from this bootstrapped dataset can only result from spurious causation due to a common history of sensory stimulation of the two considered sites. We finally used the statistics of this bootstrap estimate to express the TE estimates in units of Z score of the bootstrapped values that only contains this spurious source of causation as follows:

$$T(Y \to X) \to (T(Y \to X) - mean(T_{bootstrap}(Y \to X)) / std(T_{bootstrap}(Y \to X)) \qquad \text{(S10)}$$

The computation of LCI is exactly equal to the one described above for the TE, but considering the delayed past of the spiking activity at the sending location rather than at the receiving one.

Spatial asymmetry index of the information transfer measures

Given two recording sites A and B, we define the spatial asymmetry index as the ratio between the absolute value of the difference in TE in both directions and the maximal information in one of the two directions:

$$\frac{\left|T(A \to B) - T(B \to A)\right|}{\max(T(A \to B), T(B \to A))}. \qquad \text{(S11)}$$

The higher this ratio, the more asymmetric are the interactions, with one direction of interaction dominating the other.

COMPUTATION OF THE SPEED OF WAVE PROPAGATION

For a traveling wave with constant propagation speed *p*, the speed is related to the spatial derivative of the phase $d\varphi(x)/dx$ by

$$p = -\frac{2\pi f}{d\varphi(x)/dx}, \qquad \text{(S12)}$$

where *f* is the temporal frequency of the wave and x is the space coordinate along the direction of propagation. To assess this speed, we thus estimated phase shifts against inter-electrode distance along the directions of strongly asymmetric TE values. We first selected candidate directions of propagation by choosing each strongly asymmetric pair defined in the main text as a *reference causal pair*. For each reference causal pair, we applied the procedure described in the following paragraph.

Along the direction of the reference dominant causal pair, as shown in Fig S7, we computed the phase shifts between all pairs of electrodes having the same *receiving* electrode as the chosen reference causal pair and, as *sending* electrode, the electrodes lying on the trajectory such that the formed pair makes a maximum 45° absolute angle with respect to the inter-electrode axis of the chosen electrode pair (the electrodes lying on the gray area in Fig S7). The average phase difference between the sending electrodes and the receiving electrode was computed and stored as a function of the projected algebraic distance of the source on the axis defined by the reference pair. The receiving electrode of the reference pair, considered as the target of the wave propagation, was chosen as the origin of the x-axis. However, since the wave can propagate further to another site beyond the receiving electrode of the reference causal pair, when the receiving site was not achieving a minimum (zero) phase shift with respect to the other electrodes, but instead this minimum was achieved by the receiving site of another strongly asymmetric causal pair, this latter receiving site was chosen as the reference of the x-axis. This procedure enables to fit the phase shift as a function of propagation distance using all the directions of the propagation in all sessions. Since this function is constrained to have a zero phase shift at distance

zero, we fitted the data with a spline regression algorithm which enables to incorporate this constraint. The spline regression allows estimating the derivative of the function at the origin, and thus to compute the propagation speed at the point less susceptible to be affected by putative vanishing or overlap of traveling wave phenomena. Finally, the speed estimation was corrected taking into account that the axis of propagation is not in general aligned to axis defined by the reference causal pair. Assuming an angle θ between them, the actual speed can be estimated according to

$$p = \cos(\theta)\frac{2\pi f}{d\varphi(x)/dx}\,. \tag{S13}$$

Assuming this angle is distributed randomly between -90 and +90 degrees, the relationship is on average

$$\langle p \rangle = \frac{1}{\pi}\int_{-90^\circ}^{90^\circ} \cos(\theta)\frac{2\pi f}{d\varphi(x)/dx}\,d\theta = \frac{4f}{d\varphi(x)/dx}\,.$$

This formula was applied to compute the final speed estimate.

ANALYSIS OF VISUAL FEATURES

Orientation tuning curves

Following Ref. [7], we computed orientation tuning at each electrode from recordings of neural activity during movie stimulation by extracting the predominant orientation as the dominant gradient direction applying gradients using a Scharr operator and averaging the result across pixels belonging to the receptive field. This was done for each movie frame to evaluate the tuning curve of the multi-unit activity in the considered recording site with respect to this orientation. The preferred orientation was chosen as the one achieving the maximum of the tuning curve.

Optic flow estimation

We followed the approach of Bartels et al. [10] to estimate the optic flow of each movie stimulus. In brief, we covered the frames uniformly with square windows of 25 pixels width and 50% overlap. For each frame and each window, we detected the most similar shifted window in the next frame with the same size allowing a maximum shift of 24 pixels. The similarity between time windows was the average absolute pixel difference over the window. The difference between centers of the original window and the most similar window in the next frame was taken as an estimate of the spatial displacement vector at the location of this window for this time frame.

Movie features estimation

For each estimated receptive field, we estimated the local Time Contrast (TC) as the difference between the pixel time contrast (absolute difference of pixel luminance between successive frames, averaged over the receptive field [7]) and global time contrast (absolute difference of average frame luminance between successive frames). We also estimated the activity along the preferred orientation, denoted Orientation Activation (OA), as the squared cosine between the dominant orientation in the receptive field for this frame (estimated with Scharr operators as above) and the preferred orientation of this receptive field.

In addition we quantified the directed motion, defined as the amount of optic flow traversing the sending receptive field in the direction of the receiving receptive field. Quantitatively, if we denote by $\vec{F}_1$ the optic flow (spatial displacement) vector estimated in the square window closest to the receptive field center of the sending recording site, and θ the angle that this vector makes with the inter RF-axis of the sending and receiving sites, the directed motion writes

$$\left\|\vec{F}_1\right\|\left(\cos\theta\right)^+, \tag{S14}$$

where the "+" superscript indicates the positive part, such that the directed motion takes non-zero values only when the motion in the RF of the sending recording site is directed towards the RF of the receiving recording site.

Correlation between movie features and phase shift

For a given movie feature, we estimated the correlation between the phase shift and movie features by computing their correlation across time for each experiments. Correlation values were then averaged across all experiments to provide the final result.

Supplemental References

1. Fisher NI. Statistical Analysis of Circular Data: Cambridge University Press; 1995.
2. Montemurro MA, Rasch MJ, Murayama Y, Logothetis NK, Panzeri S. Phase-of-firing coding of natural visual stimuli in primary visual cortex. Current Biology. 2008;18:375-80.
3. Belitski A, Gretton A, Magri C, Murayama Y, Montemurro MA, Logothetis NK, et al. Low-Frequency Local Field Potentials and Spikes in Primary Visual Cortex Convey Independent Visual Information. Journal of Neuroscience. 2008;28:5696-709.
4. Kayser C, Montemurro MA, Logothetis NK, Panzeri S. Spike-phase coding boosts and stabilizes information carried by spatial and temporal spike patterns. Neuron. 2009;61:597-608.
5. Panzeri S, Treves A. Analytical estimates of limited sampling biases in different information measures. Network-Comp Neural. 1996;7(1):87-107. doi: Doi 10.1088/0954-898x/7/1/006. PubMed PMID: ISI:A1996UB77600005.
6. Magri C, Whittingstall K, Singh V, Logothetis N, Panzeri S. A toolbox for the fast information analysis of multiple-site LFP, EEG and spike train recordings. BMC Neuroscience. 2009;10:81.
7. Ince RA, Mazzoni A, Bartels A, Logothetis NK, Panzeri S. A novel test to determine the significance of neural selectivity to single and multiple potentially correlated stimulus features. Journal of neuroscience methods. 2012;210(1):49-65. Epub 2011/12/07. doi: 10.1016/j.jneumeth.2011.11.013. PubMed PMID: 22142889.
8. Schreiber T. Measuring Information Transfer. Physics Review Letters. 2000;85:461-4.
9. Besserve M, Schölkopf B, Logothetis NK, Panzeri S. Causal relationships between frequency bands of extracellular signals in visual cortex revealed by an information theoretic analysis. Journal of Computational Neuroscience. 2010;29(3):547-66. Epub 2010/04/17. doi: 10.1007/s10827-010-0236-5. PubMed PMID: 20396940; PubMed Central PMCID: PMC2978901.
10. Bartels A, Zeki S, Logothetis NK. Natural vision reveals regional specialization to local motion and to contrast-invariant, global flow in the human brain. Cerebral cortex. 2008;18(3):705-17. doi: 10.1093/cercor/bhm107. PubMed PMID: 17615246.
